# Supplementary material for: Impact of Clinical Decision Support System Assisted prevention and management for Delirium on guideline adherence and cognitive load among Intensive Care Unit nurses (CDSSD-ICU): Protocol of a multicentre, cluster randomized trial
Source: PLoS One. 2023 Nov 28;18(11):e0293950. doi: 10.1371/journal.pone.0293950 (PMC10684021; doi:10.1371/journal.pone.0293950)
Supplement: S2 File — (PDF) [file pone.0293950.s002.pdf]

S2 File: The Artificial Intelligence Assisted Prevention and Management for Delirium (*AI-AntiDelirium*)

Module 1: Delirium Assessment Tools

(Finished in preliminary work[1])

1. Delirium Assessment Tool: ICU nurses select the appropriate delirium assessment tool based on the working environment and the applicable population, including the Confusion Assessment Method for the Intensive Care Unit (CAM-ICU) and the Intensive Care Delirium Screening Checklist (ICDSC) which suggested by the Clinical Practice Guidelines for the Prevention and Management of Pain, Agitation/Sedation, Delirium, Immobility, and Sleep Disruption in Adult Patients in the ICU[2]. After the delirium assessment tool is selected, the assessment is carried out item by item according to the relevant prompts. Finally, the system will automatically determine whether the patient has delirium or not according to the evaluation results of each item (Figure 1).

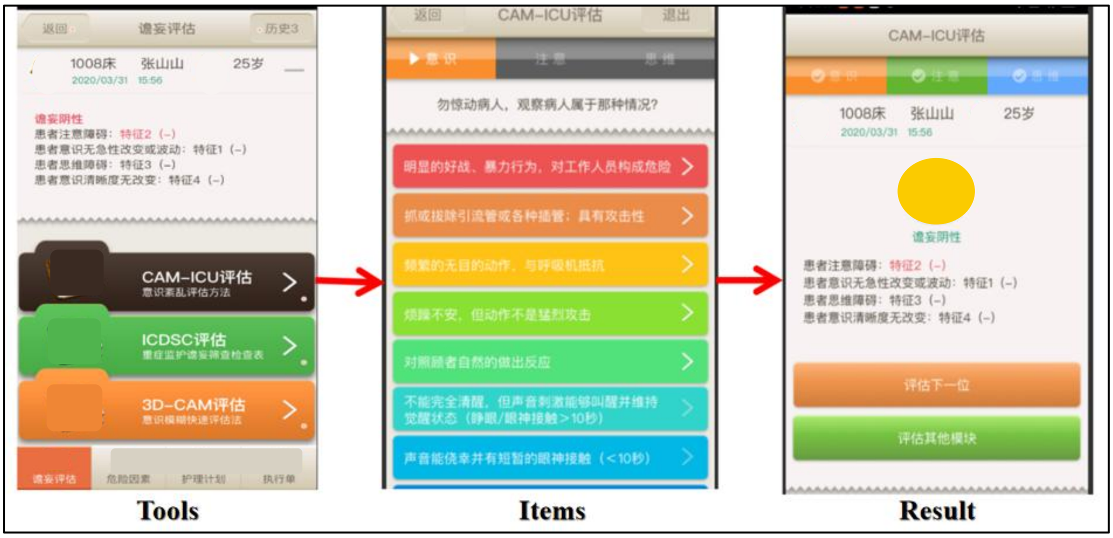

Figure 1 User Interface of Delirium Assessment Tools

2. Delirium Assessment History: Nursing staff can view the patient's previous delirium assessment information at any time. Nurses click "History" to view the patient's previous delirium assessment results (Figure 2).

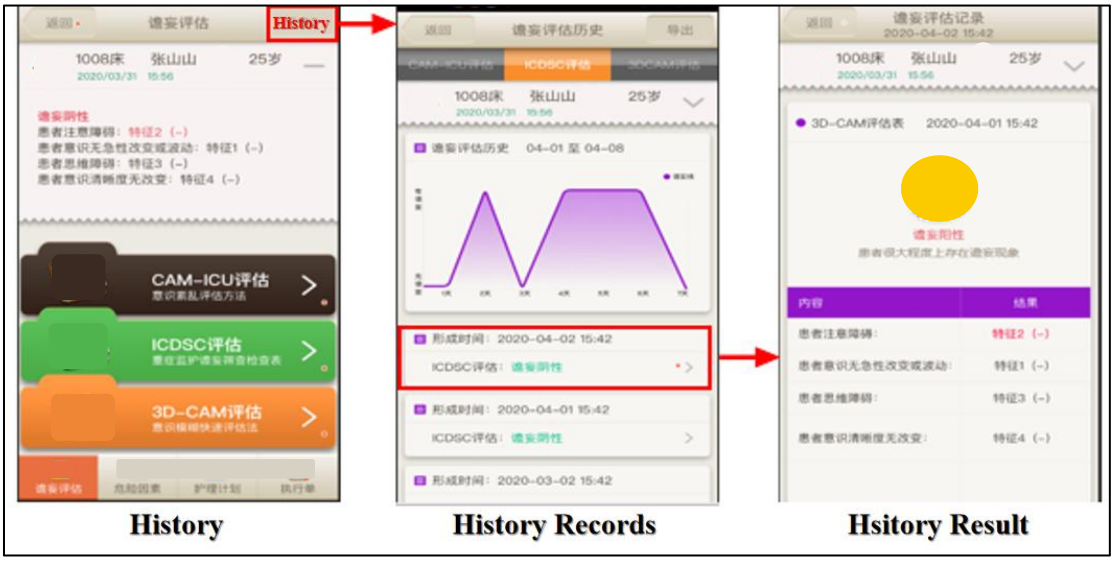

Figure 2 User Interface of Delirium Assessment History

Module 2: Risk Factors Assessment

1. ICU Delirium Risk Factor Assessment Sheet: Nursing staff enters the "ICU delirium risk factor assessment sheet" by clicking "Risk Factor Assessment" and completing the item by item. After clicking "Confirm", the *AI-AntiDelirium* will automatically display the patient's current risk factors for delirium and the predicted value of delirium (Figure 3).

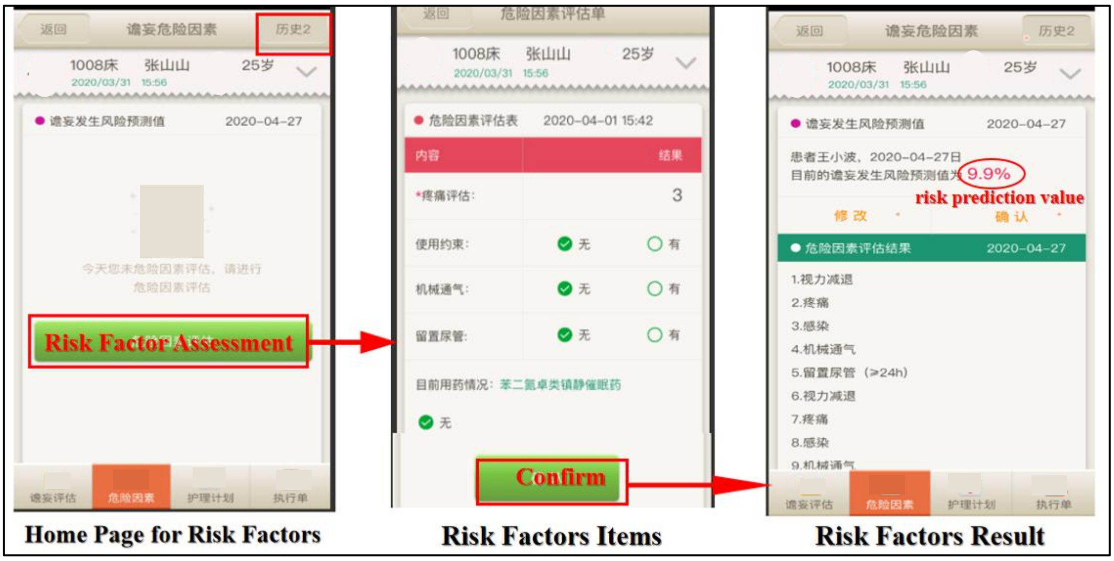

Figure 3 User Interface of the Delirium Risk Factor Assessment

2. Dynamic ICU Delirium Prediction Rule (finished in preliminary work[3]): Predictors included in the model including the APACHE-II score, history of chronic disease, sleep deprivation, use of anesthetic or sedatives, infection, indwelling

catheter, and hearing impairment. The simplified score for each predictor was 3, 3, 3, 3, 4, 8, and 9 points. The total score of delirium risk in ICU patients was calculated by adding the scores of all predictive factors. Patients were divided into low-risk (0-9 points), medium-risk (10-17 points), and high-risk groups (18-33 points). The corresponding predicted delirium rates were 2.8%, 16.8%, and 75.9%, respectively.

Example 1: After completing the "ICU Delirium Risk Factor Assessment Sheet" for Patient A, the nursing staff found that Patient A had sleep deprivation and the use of sedatives, and the predicted score for both delirium risk factors was three points, therefore the total score of delirium risk was six, indicating a low risk for Patient A and a predicted incidence of delirium of 2.8%.

Example 2: After completing the "ICU Delirium Risk Factor Assessment Sheet" for Patient B, the nursing staff found that Patient B had a history of chronic disease, sleep deprivation, use of sedatives, and indwelling catheter. The predicted scores of the four risk factors for delirium were 3, 3, 3, and 8, with a total score of 17, indicating that patient B was at medium risk for delirium, and the predicted incidence of delirium was 16.8%.

Example 3: After completing the "ICU Delirium Risk Factor Assessment Sheet" for Patient C, the nursing staff found that Patient C had sleep deprivation, infection, indwelling catheter, and hearing impairment. The predicted scores corresponding to the four risk factors for delirium were 3, 4, 8, and 9 points respectively, and the total score was 24 points, indicating that patient C was at high risk for delirium, and the predicted incidence of delirium was 75.9%.

3. Delirium Risk Factor Assessment History: Nursing staff can view the patient's previous delirium risk factor assessment information at any time. Nurses click "History" to view the patient's previous delirium risk factor assessment results (Figure 4). The history module for risk factor assessment recorded the number of risk factors for the patient, therefore, nurses can observe the increase and decrease of risk factors during nursing care. In addition, different risk factors held different risk prediction ratios, and the predicted value of the patient's risk for delirium per day was represented by a trend graph.

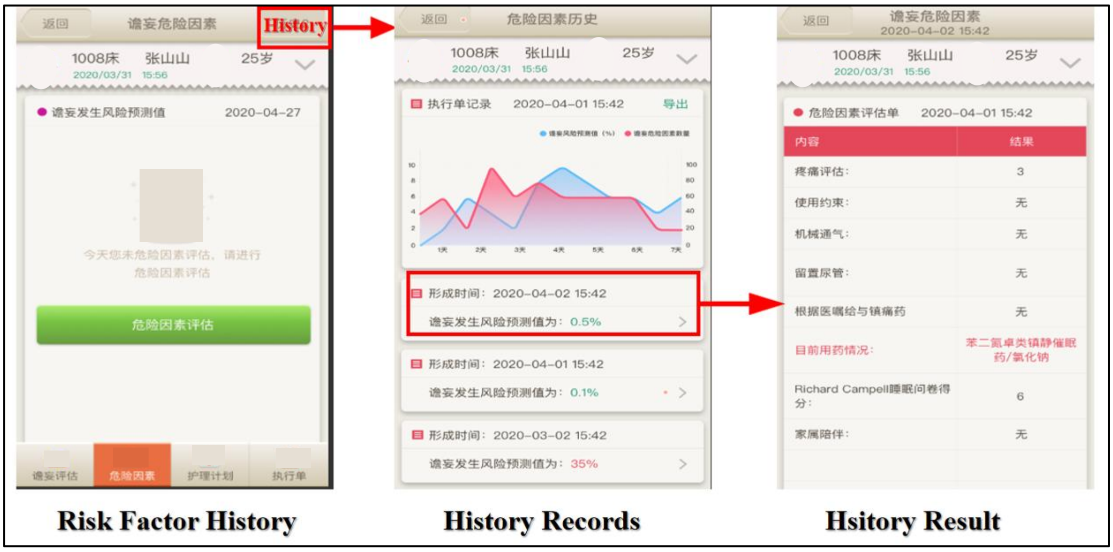

Figure 4 User Interface of Delirium Risk Factor Assessment History

Module 3: Nursing Care Plan

1. Current Nursing Care Plan: This module provides personalized delirium prevention or intervention plan according to the patient's current risk factors for delirium, and automatically displays the patient's current nursing care plan, including the formation time of the nursing plan, nursing interventions and implementation frequency (Figure 5A).

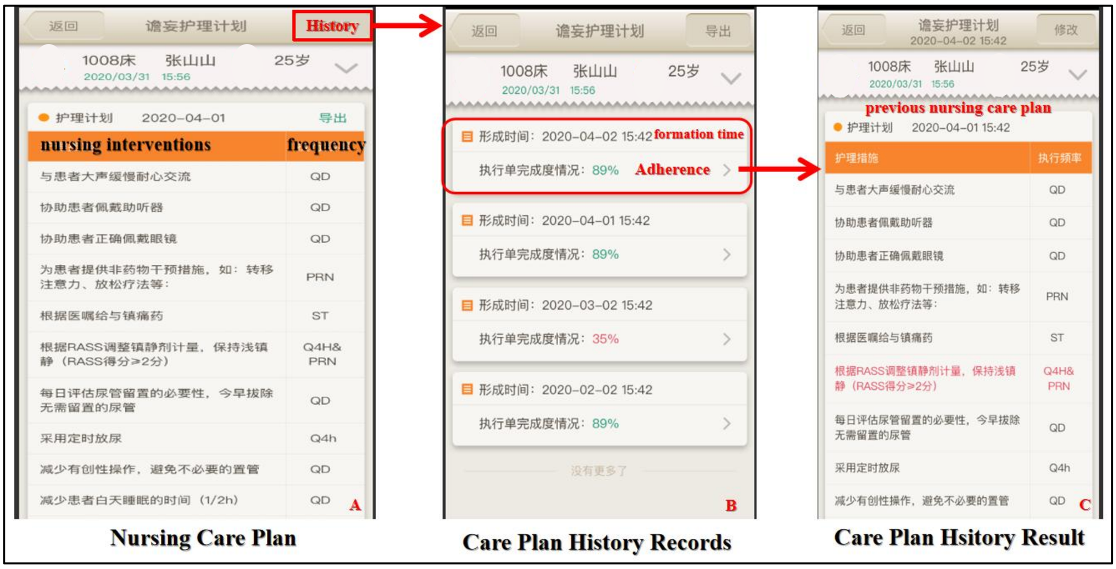

Figure 5 User Interface of Delirium Nursing Care Plan

2. Delirium Nursing Care Plan History: Nursing staff can view the patient's previous nursing care plan information at any time. Nurses click "History" to view the

patient's previous delirium nursing care plan results, including adherence, formation time, and previous nursing care plan (Figure 5B and Figure 5C). Nursing interventions not finished by the nurses would be shown in red font (Figure 5C).

Module 4: Nursing Checklist

1. Current Nursing Checklist: As shown in Figure 6A, this screen displays the execution time, nursing interventions, and executor. A circle button is placed below the executor, which clicks it when the nurse completed the intervention, and the system will automatically display the nurse's name. If the nurse does not click this button, it indicates that the nurse does not perform this intervention.

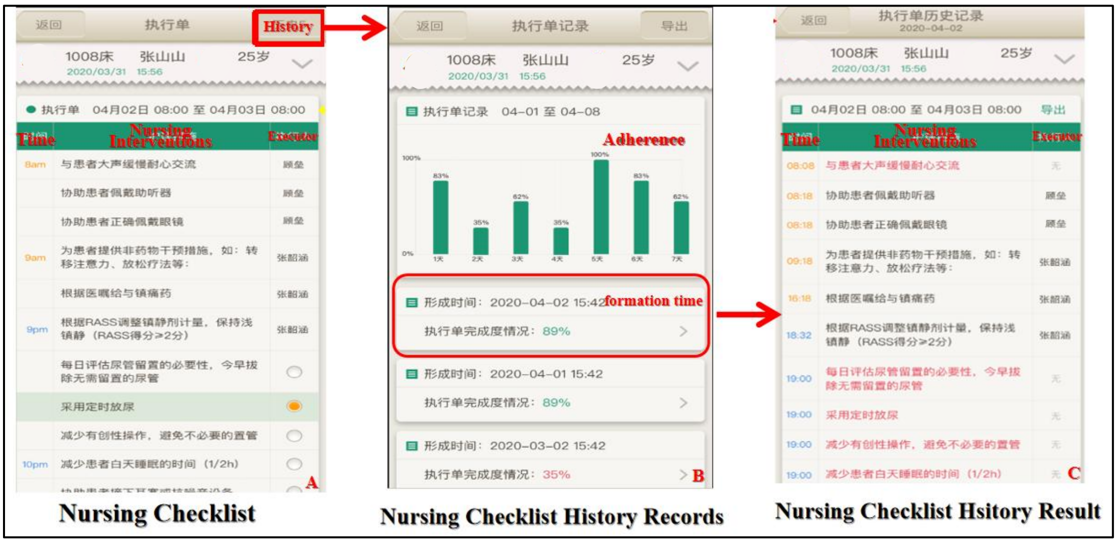

Figure 6 User Interface of Delirium Nursing Checklist

2. Nursing Checklist History: Nursing staff can view the patient's previous nursing checklist information at any time. Nurses click "History" to view the patient's previous delirium nursing checklist records (Figure 6B), including adherence and formation time. Nurses can click the specific previous nursing checklist they want to view and enter the interface in nursing checklist results (Figure 6C), including execution time, interventions, and executor. Red font interventions indicated that the nurse has unfinished care in the past but can no longer perform it (Figure 6C).

3. Nursing Checklist Reminder: The interface will remind the nurse 5 minutes in advance of the execution time of nursing interventions and display it on the screen

(Figure 7), for example, *You need to perform 3 measures for Wang-Xiaobo in 001 bed at 8:30 am,* this function will help nurses to alleviate the cognitive load.

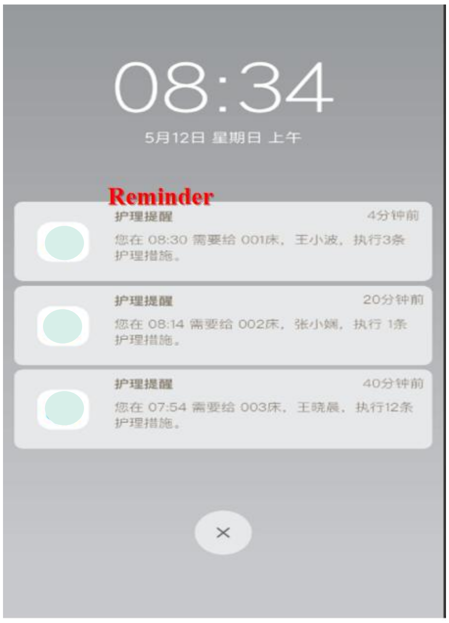

Figure 7 User Interface of Delirium Nursing Checklist Reminder

Reference

[1] Yang F，Ji M，Wu Y，et al. Delirium screening for patients in the intensive care unit: A prospective validation study of the iCAM-ICU by nurse researchers and bedside nurses in routine practice. Int J Nurs Stud， 2021， 117: 103886.

[2] Devlin J W， Skrobik Y， Gelinas C， et al. Clinical Practice Guidelines for the Prevention and Management of Pain, Agitation/Sedation, Delirium, Immobility, and Sleep Disruption in Adult Patients in the ICU. Crit Care Med， 2018， 46(9): e825-e873.

[3] Fan H，Ji M，Huang J，et al. Development and validation of a dynamic delirium prediction rule in patients admitted to the Intensive Care Units (DYNAMIC-ICU): A prospective cohort study. Int J Nurs Stud， 2019， 93: 64-73.
