# Supplementary material for: Impact of Clinical Decision Support System Assisted prevention and management for Delirium on guideline adherence and cognitive load among Intensive Care Unit nurses (CDSSD-ICU): Protocol of a multicentre, cluster randomized trial
Source: PLoS One. 2023 Nov 28;18(11):e0293950. doi: 10.1371/journal.pone.0293950 (PMC10684021; doi:10.1371/journal.pone.0293950)
Supplement: S3 File — (PDF) [file pone.0293950.s003.pdf]

S3 File: Paper-based Individual Interventions

Part One. ICU Delirium Assessment Tool

Step 1: Assessment of Awareness Level (RASS)

**Items**	**Description**	**Score**
Combative	Overtly combative, violent, immediate danger to staff	□+4
Very agitated	Pulls or removes tubes or catheters; aggressive	□+3
Agitated	Frequent nonpurposeful movement , fights ventilator	□+2
Restless	Anxious but movements not aggressive or vigorous	□+1
Alert and calm	Respond naturally to caregivers	□0
Drowsy	Not fully alert, but has sustained awakening (eye opening / eye contact) to voice (>10 seconds)	□-1
Light sedation	Briefly awakens while eye contact to voice (<10 seconds)	□-2
Moderate sedation	Movement or eye opening to voice (but no eye contact)	□-3
**If RASS ≥ -3, proceed to Step 2: CAM-ICU Assessment**		
Deep sedation	No response to voice, but movement or eye opening to physical stimulation	□-4
Unarousable	No response to voice or physical stimulation	□-5
**If RASS = -4 or -5, stop (patient is unconscious) and reassess later**		
The patient's final RASS score ______ Whether to enter the second step: □Yes □No		

Step 2: Confusion Assessment Sheet for ICU Patients (CAM-ICU)

**Feature 1: Acute Change or Fluctuating Course of Mental Status**	**Positive standard**	If positive, tick √ here
▪ Is there an acute change from mental status baseline? **OR**<br>▪ Has the patient's mental status fluctuated during the past 24 hours?	The answer to any question is "yes" →	
**Feature 2: Inattention**		
**Number-based attention**<br>▪ "Squeeze my hand when I say the number '8' ."<br>Read the following sequence of letters:<br>**6 8 5 9 8 3 8 8 4 7**<br>**ERRORS: No squeeze with "8" & Squeeze on number other than 8**	Number of errors>2 →	
**Feature 3: Altered Level of Consciousness**		
▪ Current RASS level	RASS is not "0" →	
**Feature 4: Disorganized Thinking**		
1. Will a stone float on water?<br>2. Are there fish in the sea?<br>3. Does one pound weigh more than two?<br>4. Can you use a hammer to pound a nail?<br>**Command:**<br>"Hold up this many fingers" (Hold up 2 fingers)<br>"Now do the same thing with the other hand" (Do not demonstrate)<br>**OR** "Add one more finger" (If patient unable to move both arms)	Total number of errors > 1 →	
**CAM-ICU Positive=**<br>**Feature 1(+) and Feature 2 (+) and Feature 3 (+) or Feature 4 (+)**	Yes →<br>No →	Positive<br>Negative

Part Two. Risk Factors Assessment

Risk Factors	Items	Evaluation Rules
A1<br>**Hearing impairment**	Hearing loss<br>☐ 1. Normal<br>☐ 2. Mild to moderate<br>☐ 3. Severe	○ 1. Normal (can hear and repeat normal voice at a distance of 1m without difficulty)<br>○ 2. Mild to moderate (there is hearing difficulty in some environments, the speaker needs to increase the volume or slow down the speed of speech so that the patient can hear and repeat the raised voice at a distance of 1m)<br>○ 3. Severe (can't hear the voice at all)<br>**When the hearing loss is severe, it suggests that the patient has a risk factor for delirium — "hearing loss", and requires further intervention.**
A2<br>**Vision impairment**	Vision loss<br>☐ 1. Normal<br>☐ 2. Mild to moderate<br>☐ 3. Severe	○ 1. Normal (uncorrected visual acuity ≥ 5.0 , tiny details can be seen, including general printed newspapers / books)<br>○ 2. Mild to moderate (naked eye vision is 4.5-4.9 ; objects can be identified; large prints can be read)<br>○ 3. Severe (uncorrected visual acuity ≤ 4.5 ; unable to see objects or difficult to identify objects)<br>**When the vision loss is severe, it suggests that the patient has a risk factor for delirium — "visual loss", and requires further intervention.**
A3<br>**Pain**	Pain Score: __________	**Assess patient pain according to the Pain Numerical Scale (NRS)**<br>Evaluation criteria: Patients were asked to describe the pain intensity with 0-10, 0 being no pain, 1-3 being mild pain, 4-7 being moderate pain, >7 being severe pain, and 10 being severe pain.<br>**When the pain score is not 0 , it indicates that the patient has the risk factor of delirium — "pain", and needs further intervention.**
A4<br>**Use of anaesthetic or sedatives**	**Current medication status (multiple choices)**<br>**A4.1 Benzodiazepine sedative-hypnotics**<br>□No<br>□ Diazepam<br>□ Midazolam<br>□ lorazepam<br>□ Chlordiazepoxide<br>□ Alprazolam<br>□ Estazolam<br>□Clonazepam<br>□Other _____<br><br>**A 4.2 Analgesic and sedative drugs**<br>□No<br>□ Dexmedetomidine<br>□Propofol<br>□ Morphine<br>□ Pethidine<br>□ Bunarizine<br>□Other _____	**When a patient is on either sedative, it suggests that the patient has a risk factor for delirium — "sedative therapy", and requires further intervention.**

	**A 4.3 Anesthetics**<br>□No<br>□ Remifentanil<br>□ Sufentanil<br>□ Midazolam<br>□Li Yuexi<br>□ Tramadol<br>□ Wan Wen<br>□ Flumazenil<br>□Naloxone<br>□ Baquting<br>□A strong dragon<br>□Ephedrine<br>□Other _____<br><br>**A 4.4 Anticonvulsants**<br>□No<br>□ Phenobarbital<br>□ Chloral hydrate<br>□ Magnesium sulfate injection<br>□Other _____<br><br>**A 4.5 Antipsychotics**<br>□No<br>□ Chlorpromazine<br>□ Haloperidol<br>□ Olanzapine<br>□Other _____<br><br>**A 4.6 Anticholinergics**<br>□No<br>□ Atropine<br>□ Glycopyrrolate<br>□ Penehyclidine<br>□ Scopolamine<br>□ Anisodamine<br>□Other _____<br><br>**A 4.7 Other psychoactive drugs**	
A5<br>**Mechanical Ventilation**	Invasive ventilator support therapy<br>☐ 1. Yes<br>☐ 2. No	**When the patient was treated with invasive ventilator support, it was suggested that the patient had the risk factor of delirium — "Mechanical Ventilation", and further intervention was required.**
A6<br>**Indwelling Catheter**	Indwelling catheter<br>☐ 1. Yes<br>☐ 2. No	**When a patient has an indwelling catheter, it is suggested that the patient has a risk factor for delirium — "indwelling catheter", and requires further intervention.**
A7<br>**Infect**	A7.1 Infection<br>☐ 1. Yes<br>☐ 2. No<br><br>A7.2 Type of infection<br>☐ 1. Respiratory infection<br>☐ 2. Urinary tract infection<br>☐ 3. Wound infection	**When the patient has an axillary temperature ≥ 37.5°C and the absorption of heat after surgery is excluded, or the culture of blood, urine and sputum is positive, it indicates that the patient has the risk factor of delirium— "infection" , and needs further intervention.**

	☐4. Other _____	
A8<br>**Immobility**	**Does the patient have any of the following:**<br>**(single choice or multiple choice)**<br>☐ 1. None<br>☐ 2. Use constraints<br>☐ 3. Treated with mechanical ventilation<br>☐ 4. Use sedatives<br>☐ 5. Active bleeding<br>☐ 6. Spinal cord injury<br>☐ 7. Open Lumbar Drainage<br>☐ 8. Extracranial Ventricular Drainage<br>☐ 9. Unstable fracture<br>☐ 10. Active myocardial infarction<br>☐ 11. Arrhythmia<br>☐ 12. Respiratory distress<br>☐ 13. Restlessness (RASS score ≥2)<br>☐ 14. Systolic blood pressure <90mmhg or >180mmhg<br>☐ 15. Heart rate <60 beats/min or >130 beats/min<br>☐ 16. Respiratory rate <5/min or >40/min<br>☐ 17. Pulse oximetry <88%<br>☐ 18. Physician ordered bed rest/no activity<br>☐ 19. Use of vasopressors (eg, dopamine, dobutamine, epinephrine, norepinephrine, vasopressin)	**When "none" is selected, it indicates that the patient can perform appropriate early activities ; when any of the other options is selected , it indicates that the patient cannot perform early activities, and there is a risk factor for delirium— "Immobility", which requires further intervention.**
A9<br>**Sleep disorders**	**Richard Campbell Sleep Questionnaire Score : __________**	**Richard Campbell Sleep Questionnaire**<br>(0 points - not good; 100 points - good)<br>○ Sleep depth: _____ points<br>○ Easy to fall asleep: _____ points<br>○ Easy to wake up during sleep: _____ points<br>○ Going back to sleep after waking up: _____ points<br>○ Overall quality of sleep: _____ points<br>**When a Richard Campbell Sleep Questionnaire score ≤ 25 indicates the presence of a delirium risk factor —"Sleep disorders", further intervention is required.**
A10<br>**No family members visit**	Accompanying family members<br>**☐ 1. Yes**<br>**☐ 2. None**	When selecting "none" indicates that there is a risk factor for delirium — "No family members visit", further intervention is required.

Part Three. Prevention and Management Interventions for Intensive Care Unit Delirium

Risk factors	IF	AND	Nursing Interventions Classification	Nursing Interventions	Frequency
			**Usual care**	Place calendar and clock with the correct date and time in the ICU	QD
				Inform the patient the current time and place in detail	QD
				Turn off the ICU headlights and lower the brightness of corridor lights	qn
				Reduce the alarm sound of medical equipment	qn
				Lower staff's communication voice	qn
				Reschedule medications and procedures to reduce disturbed sleep of patients	qn
Hearing impairment	No hearing aids		Treatment and nursing care	Speak loudly, slowly and patiently with the patient	QD
			Health guidance	Advice family members to buy hearing aids for the patient	Visiting hours
	Have hearing aids	at home	Health guidance	Remind family members to bring hearing aids to the ICU during the next visit	Visiting hours
		in the ICU	Treatment and nursing care	Assist patient to wear hearing aids correctly	QD
			Treatment and nursing care	Assist patient to take hearing aids off correctly	QD
Visual impairment	No eye glasses		Health guidance	Advice family members to buy eye glasses for the patient	Visiting hours
	Have eye glasses	at home	Health guidance	Remind family members to bring eye glasses to the ICU during the next visit	Visiting hours
		in the ICU	Treatment and nursing care	Assist patient to wear eye glasses correctly	QD
			Treatment and nursing care	Assist patient to put eye glasses off correctly	QD
Pain			Treatment and nursing care	Provide non-pharmacological interventions, such as distraction and relaxation therapy	PRN
			Treatment and nursing care	Use analgesics according to the doctor's order	st

Table S1. Prevention and Management Interventions for Intensive Care Unit Delirium (Continued)

Risk factors	IF	AND	Nursing Interventions Classification	Nursing Interventions	Frequency
Use of anaesthetic or sedatives			Pharmacotherapy and nursing	Adjust the sedative dose according to RASS and maintain light sedation (RASS score ≥-2)	Q4H & PRN
	Dexmedetomidine was not used	Propofol was not used	Pharmacotherapy and nursing	Remind doctors to use dexmedetomidine or propofol	QD
	Spontaneous awakening trial (SAT) Safety Screen: PASS.		Treatment and nursing care	Implementation of SAT: Reduce the daily dose of sedatives, make fully awake until the patient was able to answer a few simple questions or do some simple command action (i.e. blinking, finger), and then trained doctors or nurses would readjust dosage of sedative drugs to achieve expected sedation level.	QD
	SAT Safety Screen: Failure.		Illness monitoring	Observe patient's respiratory status	PRN
	Have performed SAT	SAT Safety Screen Failure	Pharmacotherapy and nursing	Restart sedation at 50% of prior dose	QD
Mechanical Ventilation	SBT Safety Screen: PASS.	Doctor's advice: perform SBT	Treatment and nursing care	Conduct SBT according to the doctor's order	QD
		No doctor's advice about performing SBT	Treatment and nursing care	Advise doctors to consider SBTs	st
	SBT Safety Screen: Failure		Illness monitoring	Observe the patient's respiratory status	PRN
Indwelling catheter			Treatment and nursing care	Remove the catheter as soon as possible	QD
			Treatment and nursing care	Conduct timed urination for the patient	Q4h
Infection			Treatment and nursing care	Reduce invasive operations and avoid unnecessary catheterization	QD
	Respiratory tract infection		Illness monitoring	Observe the patient's respiratory status	PRN
	Wound infection		Treatment and nursing care	Change dressing for the wound in time to keep the wound clean	QD
	Urinary tract infection		Treatment and nursing care	Advise doctors to remove catheter as soon as possible	st

Table S1. Prevention and Management Interventions for Intensive Care Unit Delirium (Continued)

Risk factors	IF	AND	Nursing Interventions Classification	Nursing Interventions	Frequency
Immobility	Have activity contraindications		Rest and exercise	Level0: patients are advised to rest in bed and not to be active	st
	No activity contraindications	RASS =-3 or -4 or -5	Rest and exercise	Level1: help patients to do passive range-of-motion exercises, 10 times for each joint	QD
		RASS=**-2** or **-1**or **0** or **+1**）	Rest and exercise	Level2: patients are advised to do the active range-of-motion exercises on the bed (10-20 minutes)	QD
			Rest and exercise	Level3: help patients sit on the bedside for 20 minutes	QD
			Rest and exercise	Level4: Assist the patient to stand still at the bedside for 5-10 mins	QD
			Rest and exercise	Level5: Assist patients to walk along the aisle for 5-10 minutes	QD
Sleep disorders（RCSQ ≤25）			Rest and exercise	Reduce the time of sleep during the day (< 1/2h)	QD
	Daytime: 8am-8pm		Treatment and nursing care	Assist patients to remove earplugs or anti-noise equipment	QD
			Treatment and nursing care	Assist patients to remove eye mask	QD
	Nighttime: 8pm-8am		Treatment and nursing care	Assist patients to wear earplugs or anti-noise equipment	QD
			Treatment and nursing care	Assist patients to wear eye mask	QD
No family members visit			Social support	Family visits are encouraged	Visiting hours
Delirium（CAM-ICU (+) or ICDSC≥4）			Psychological support	Communicate with patients and comfort them with care in order to lower their panic and anxiety	PRN
			Social support	Encourage family members to visit patients and help patients with orientation training	Visiting hours
			Health guidance	Conduct propaganda to patient's family members, such as, symptoms and signs of delirium, treatment nursing interventions	Visiting hours
	No use of non-dexmedetomidine sedatives	RASS=2 or 3 or 4	Pharmacotherapy and nursing	Advise doctors to administer non-benzodiazepine sedatives such as dexmedeidine to lower the risk of delirium	st
	Invasive MV		Treatment and nursing care	Enforce protective restraint	PRN
