# Supplementary material for: Impact of Clinical Decision Support System Assisted prevention and management for Delirium on guideline adherence and cognitive load among Intensive Care Unit nurses (CDSSD-ICU): Protocol of a multicentre, cluster randomized trial
Source: PLoS One. 2023 Nov 28;18(11):e0293950. doi: 10.1371/journal.pone.0293950 (PMC10684021; doi:10.1371/journal.pone.0293950)
Supplement: S4 File — (PDF) [file pone.0293950.s004.pdf]

S4 File. Research project submitted to the ethics committee (English)

Title: The role and the mechanism of cognitive load in influencing the nursing adherence to delirium intervention among ICU nurses

PhD student: Shan Zhang

Advisor: Prof. Dr. Ying Wu

Institution: Capital Medical University

Summary

High cognitive load of nurses is a common problem in intensive care unit, which is associated with nurses' poor performance of activities, as well as related to poor patient outcomes. It remains unclear whether **A**rtificial **I**ntelligence **A**ssisted Preve**nti**on and Management for **Delirium** (*AI-AntiDelirium***)** results in reduction of cognitive load and enhances adherence to delirium intervention in ICU nurses. We aim to assess the effectiveness of *AI-AntiDelirium* on cognitive load and adherence to delirium interventions of ICU nurses and the impact of ICU delirium. This is a multicentre, prospective, cluster randomised controlled clinical trial comparing the impact of *AI-AntiDelirium* and PADIS guideline for ICU nurses. A total of six ICUs in two hospital will be randomised in a 1:1 ratio to receive either *AI-AntiDelirium* (intervention group) or PADIS guideline (control group), with target sample size of 78 ICU nurses. The primary endpoint will be the adherence to delirium interventions. The secondary endpoints will be the cognitive load which measured by an instrument for measuring different types of cognitive load (MDT-CL). The endpoints of patients included incidence and duration of ICU delirium, length of ICU and in-hospital stay, ICU and in-hospital mortality. Outcome assessments will be conducted by investigators who blind to group assignment. The repeated measures analysis of variance (RMANOVA) will be performed to detect the difference in adherence and cognitive load between groups. Generalized estimation equation will be performed to

test the difference in patient outcomes.

Background

Cognitive load refers to the total amount of cognitive resources that a person needs to process cognitive activities[1], and is composed of three different types: intrinsic CL, extraneous CL and germane CL[2,3]. Higher CL, which exceeds one's working memory resource capacity, has been identified as one of the most important problems in providing intensive care[4,5]. It negatively impacts nurses and their patients[4], which can lead to irritability, memory impairments and mental fatigue of nurses[6,7], is detrimental to performance of activities, reduced learning capacity for acquiring knowledge[8,9], and also results in poor patient outcomes and compromises patient safety[6]. The more complicated are the activities performed by nurses, the higher the CL are required, which will hinder the adherence to implement nursing activities[10,11].

Nursing care in intensive care units (ICU) is characterized by extremely demanding caseloads, performance of complicated activities and making complex decisions[4]. One example is the complex care required in ICU delirium prevention and management interventions. ICU delirium is a common complication of ICU patients with an high incidence of 70% to 87%[12,13], and associated with longer hospital length of stay (LOS) and increased mortality[13-15]. Therefore, the Clinical Practice Guidelines for the Prevention and Management of Pain, Agitation/Sedation, Delirium, Immobility, and Sleep Disruption in Adult Patients in the ICU (PADIS guideline) recommend use of the ABCDEF bundle as a way to prevent and manage ICU delirium[16-18], which focuses on eliminating ICU delirium risk factors[18-20]. However, adherence to the ABCDEF bundle is sub-optimal in routine clinical care[21,22]. Previous studies have demonstrated that various barriers may hinder the adherence to

implement the bundle, such as heavy workload on nursing care records[23], complexity algorithm of assessment tools[24,25], struggle to collect and remember numerous risk factors through multiple channels[26], lack of knowledge about ICU delirium[27]. Working on complicated activities results in lowered speed for receiving and processing information, decreased capacity of working memory and requires higher cognitive load (CL)[28,29].

Considering the negative consequence of high CL from delirium care, it is important to provide ICU nurses with the aid of a tool that can reduce CL of nursing care in ICU delirium. With the rapid development of information technology, clinical decision support system (CDSS) has been widely used in most hospitals in worldwide for disease assessment, management and record[11,30-32]. The CDSS play an important role in clinical nursing care, and also commonly used in cognitive psychology[33,34]. It can collect, sort, classify and establish logical relationship of the patient information, and also make use of alert, information feedback to provide decision support during disease diagnosis, treatment and nursing activities[35,36]. Several studies have shown that CDSS can help medical staff to recall less clinical information, which lead to a significant reduction in CL and improvement the adherence to implement nursing interventions[37,38].

Therefore, we developed an **A**rtificial **I**ntelligence **A**ssisted Preve**nti**on and Management for **Delirium** (***AI-AntiDelirium***) which including ABCDEF bundle intervention, risk factors and assessment tools of ICU delirium. The aims of this study are to assess the effectiveness of *AI-AntiDelirium* on adherence and cognitive load of ICU nurses, as well as clinical outcomes related to ICU delirium.

Methods

The protocol is developed according to the Standard Protocol Items: Recommendations for Interventional Trials (SPIRIT)[39].

Study design and setting

The *AI-AntiDelirium* to improve adherence to delirium intervention of ICU nurses is a multicenter, cluster randomized controlled trial (RCT), six ICUs with higher incidence of ICU delirium in two tertiary hospitals are selected, which represent three types of ICUs (2 Surgical ICU [SICU], 2 Respiratory ICU [RICU] and 2 Cardiac ICU [CICU]) as well as the variety of the samples, and the promise of ICU staff to improve the quality of care in ICU delirium. A tertiary hospital is a 1000-bed university-affiliated teaching hospital, with 42 adult ICU beds in the 3 participating ICUs. B tertiary hospital has 730 beds and 3 eligible ICUs of 46 beds, which the annual admission rate is about 850 patients. They have fully implemented hospital informatization. The study will be conducted during the period 2022 - 2023. Figure 1 shows the design of the study.

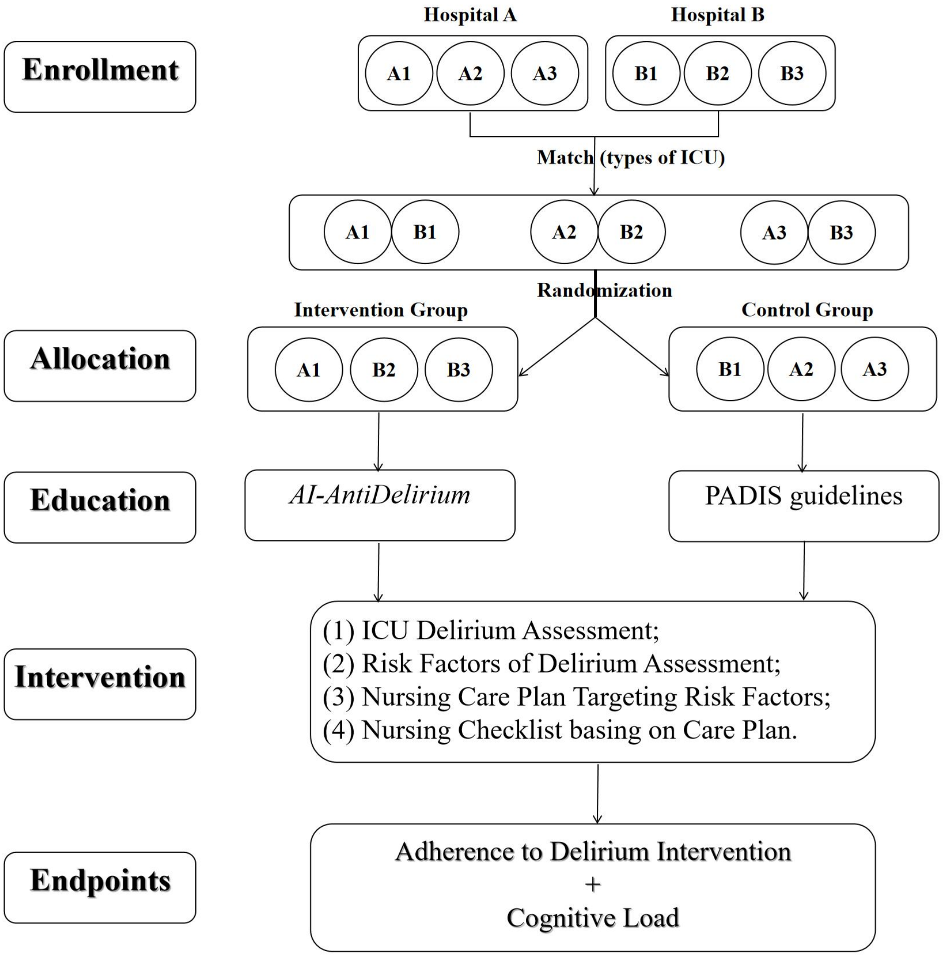

Figure 1 study design

AI-AntiDelirium, Artificial Intelligence Assisted Prevention and Management for Delirium;

PADIS guideline, Clinical Practice Guidelines for the Prevention and Management of Pain, Agitation/Sedation, Delirium, Immobility, and Sleep Disruption in Adult Patients in the ICU;

Participants

Registered Nurses (RN) who worked in included ICUs were all recruited. Nurses were eligible for the study if they (1) had a minimum of 1 year experience in intensive care; (2) worked full-time in the unit; and (3) consented to participate in this study. Nurses who were on study programs outside the hospitals or on leave for various reasons during the study period were excluded.

Intervention Groups

Prior to the study, an educational program is delivered by researchers, including knowledge regarding ICU delirium risk factors, assessment tools (Confusion Assessment Method for the Intensive Care Unit [CAM-ICU], Intensive Care Delirium Screening Checklist [ICDSC]), and ABCDEF bundle interventions. Except that, eligible ICU nurses are trained with how to operate the *AI-AntiDelirium*. We developed the *AI-AntiDelirium*, which included the four main modules: ICU delirium assessment module (Figure 1A), risk factors assessment module (Figure 1B), nursing care plan module (Figure 1C) and nursing checklist module (Figure 1D).

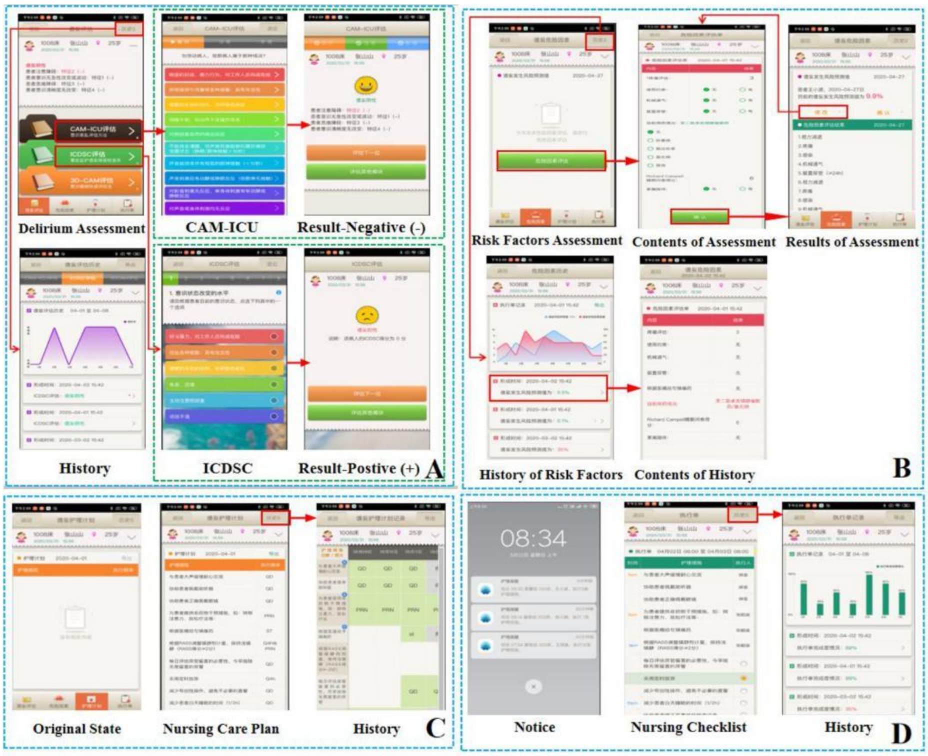

Figure 1. Modules of Application

A: ICU delirium assessment tools and results; B: risk factors assessment and predictive risk value; C: nursing care plan; D: nursing checklist

ICU nurses in the intervention group will provide ICU delirium nursing care based on *AI-AntiDelirium*. Each day, the nurse will assess ICU patients with the assistance of *AI-AntiDelirium*, which can recognize ICU delirium, automatically

present risk factors and tailor evidence-based prevention or management strategies of ICU delirium. During the study, the nurse who receive ICU delirium training clicks the icon of *AI-AntiDelirium* on the Personal Digital Assistant (PDA) to enter the system.

a．**Register**: click the icon of register → enter the user name → enter the password → choose the hospital and department → submit;

b．Log in: enter the user name and password → log in.

c．**Add the patient**: click the button "+" → enter the medical record No. → enter patient name → enter patient gender → enter patient age → enter patient bed number → enter patient admission time.

d．**Diagnose ICU delirium**: click the button of delirium assessment → select a delirium assessment tool (CAM-ICU or ICDSC) → complete the item one by one according to the prompts → the *AI-AntiDelirium* will automatically display whether the patient has delirium or not.

e．**Identify patients ICU delirium risk factors:** click the button of "risk factors assessment" → complete a brief risk factor assessment evaluation list, including three categories, predisposing (e.g. hearing impairment, vision impairment), disease-related (e.g. infection, pain), iatrogenic and environmental factors (e.g. mechanical ventilation, sedative use) → the *AI-AntiDelirium* will automatically report personalized risk factors of the patient → the *AI-AntiDelirium* will automatically show the risk prediction value in developing delirium according to a dynamic ICU delirium prediction rule[40].

f. **Confirm nursing care plan:** click the button of "nursing care plan" → the *AI-AntiDelirium* will automatically present individual ICU delirium prevention or management care plan according to the result of risk factors assessment → the ICU

nurse checks and confirms that all interventions for the patient are appropriate and feasible.

g. **Implement individual, risk factors targeting interventions:** click the button of “nursing checklist” → the *AI-AntiDelirium* will automatically automatically display the specific execution time of each intervention basing on the results of nursing care plan → the nurse implements individual interventions during the whole shift and records reasons why the patient do not receive these interventions → the nurse clicks on the measure after implements it → the *AI-AntiDelirium* automatically record the nurse's name and the time of execution.

Control group

Prior to the study, an educational program related to ICU delirium is delivered by researchers, the content are same to intervention group. In addition, nurses are trained with the usage of PADIS guideline. During the study, nurses will provide ICU delirium nursing care according to PADIS guideline, including ICU delirium risk factors, assessment tools (CAM-ICU, ICDSC), and ABCDEF bundle intervention.

a. **Diagnose ICU delirium:** The nurse chooses one instrument (CAM-ICU or ICDSC) to assess ICU delirium occurrence and completes the item one by one. The nurse manually records the assignment score of each item, and calculates the final total score, and then they judge whether the patient develops delirium or not according to the rule of assessment tool.

b. **Identify patients ICU delirium risk factors:** The ICU nurse fills out a brief risk factor assessment for delirium (the content same to intervention group). The nurse makes decision by themselves about patient's current risk factors according to the risk factors assessment results and manually calculates the patient's current risk of developing ICU delirium according to the dynamic ICU delirium prediction rule.

c. **Implement individual, risk factors targeting interventions**: The nurse extracts nursing intervention from the PADIS guideline according to the result of patient' specific risk factors, and records the intervention in nursing care records, and implements appropriate subset of interventions.

Outcomes

Endpoint assessment will implement by trained research staffs who will not involve in the clinical nursing care of patients.

The primary outcome is **adherence to the intervention, which** is defined as the adherence of nurses to implement ICU delirium and risk factors assessment, delirium prevention and management interventions every day. The reasons for non-adherence will record daily by the intervention staff.

a. Adherence to ICU delirium assessment was calculated by dividing the number of actions to number of theoretical assessment. The theoretical ICU delirium assessment for each nurse per day was defined as twice daily multiply the number of patients cared by the nurse.
b. Adherence to ICU delirium risk factors assessment was calculated by dividing the number of actions to number of theoretical risk factors assessment. The theoretical risk factors assessment for each nurse per day was defined as once daily multiply the number of patients cared by the nurse.
c. Adherence to ICU delirium prevention or management intervention was calculated by dividing the number of actions to number of theoretical interventions implementation

Secondary endpoints will include cognitive load of nurses, **which** defined as the total amount of limited cognitive resources that a person is occupied during a cognitive task[1]. Cognitive load was assessed by means of an instrument for measuring

different types of cognitive load (MDT-CL) with **ten-item**, which had been successfully used to distinguish different types of cognitive load[41]. Each item score from 0 to 10, the higher of the score, the higher of the cognitive load. Cronbach's α of Chinese version MDT-CL was 0.818, cronbach's α values in measurement of intrinsic CL (Items 1, 2, and 3) was 0.879; cronbach's α values in measurement of extraneous CL (Items 4, 5, and 6) was 0.878; cronbach's α values in measurement of germane CL (Items 7, 8, 9, and 10) was 0.946.

Participant timeline

Enrollment and data collection started in November 2022. The recruitment will continue until the target population (78 nurses) is enrolled, which is expected to be ended in June 2023. And afterward, the data analysis will be implemented for publications.

Sample size

The aim of the cluster RCT is to improve the adherence to delirium intervention of ICU nurses by using the *AI-AntiDelirium* compared to the PADIS guideline. We expect that adherence to delirium intervention in the *AI-Antidelirium* group is 80% and PADIS guideline group is 50%[42], suggesting that *AI-Antidelirium* has clinical significance in improving the adherence to delirium intervention of ICU nurses. Sample size calculations showed that 12 nurses in each cluster will provide 80% power and a two-sided significance level (α) of 0.05, with an intracluster (within-unit) correlation of 0.002[43]. In addition, taking into account possible dropout rate of 10%, we plan to enroll 13 nurses in each ICU, the final sample size is 78 (13*6) nurses.

Recruitment

All recruitment will carry out by trained research staffs who will not involve in the intervention and blind to the nurses' group assignments. Researchers will screen

nurses daily base on the inclusion and exclusion criteria. Written informed consent will be obtained from all eligible nurses. To retain more nurses, research staffs will explain the benefit for nurses if this study successfully implement.

Randomisation

The risk of between-group contamination is reduced by cluster randomisation, ICUs will be randomised 1:1 to receive either *AI-AntiDelirium* care or PADIS guideline. We plan to recruit 6 ICUs in two hospitals, and matching the types of ICU (considering the different types of ICU nursing process, workload and patients illness severity and other factors which will affect the nurses' adherence). The allocation sequence is based on computer-generated random numbers, which is performed by a statistician who is independent of data analyses and not involved in data collection. In order to ensure the allocation concealment, the statistician will inform the each ICUs' allocation code to the study coordinator. And then, the study coordinator will inform the ICU nurses about which group they are allocated. All eligible nurses are recruited until the sample size is enough.

Blinding

Investigators who will enroll participants are are not made aware of the randomisation list and patients are kept unaware of their assignment. But the nurses who implement strategies are not be possible to blind the allocation due to the nature of the intervention. Baseline data and endpoints measures will be collected by data collectors or outcome assessors who have no role in the intervention and blind to the allocations.

Data collection

As shown in Table 1, prior to the study, uniform training will be delivered by research staff to data collectors. All study data are anonymized and treated confidentially.

Informed consent will be obtained from all ICU nurses prior to study participation. After that, basic demographic data of ICU nurses are collected, including: age, gender, marital status, education background, departments where they worked, years of ICU experience, professional title, baseline CL and level of knowledge. Level of knowledge refers to the nurses' knowledge of ICU delirium assessment tools, risk factors, prevention and intervention acquired through learning, or clinical practice, and measured using the Questionnaire of ICU delirium knowledge. A 20-item multiple-choice knowledge questionnaire was newly developed, with content validity was 0.96, overall cronbach's α values of the questionnaire was 0.814[44]. The higher of the score, the higher knowledge regarding ICU delirium. During the study, CL and adherence to interventions of ICU nurses are recorded each day at the end of the shift. Data are stored on electronic file powered by a data manager.

Table 1 ICU nurses' adherence to delirium intervention improvement protocol schedule of forms and procedures

Assessment content \ Assessment time	Recruitment	Baseline assessment	Daily assessment	End of the study
**Enrollment**				
Eligibility screen	×			
Informed consent	×			
**Baseline assessment**				
Age		×		
Gender		×		
Marital status		×		
Education background		×		

Ethnicity	×		
Department	×		
Years of ICU experience	×		
Professional Title	×		
Level of knowledge about ICU delirium	×		
**Allocation**	×		
**Interventions**			
AI-AntiDelirium		×	
Step1: ICU delirium assessment		×	
Step2: Risk factors assessment		×	
Step3: Nursing care plan		×	
Step4: Nursing Checklist		×	
**Paper-based PADIS guideline**		×	
Step1: ICU delirium assessment		×	
Step2: Risk factors assessment		×	
Step3: Nursing care plan		×	
Step4: Nursing Checklist		×	
**Outcomes**			
Adherence to ICU delirium assessment		×	×
Adherence to risk factors assessment		×	×
Adherence to delirium prevention or management intervention		×	×
Cognitive load	×	×	×

Data management

All data will be collected on a printed, precoded forms that will be double entered into an electronic data base and go though extensive error checking and data completeness. The ICUs involved in the study only have access to their own data. We will perform the following processes to promote data quality: (1) Before the study, all investigators and data collectors will be trained to master the procedures of data collection; (2) All participate nurses will be trained to apply the tools, but different group nurses will attend different education session, intervention group nurses will learn how to use the *AI-AntiDelirium*, control group nurses will learn how to use the PADIS guideline; (3) Data cleaning and reviewing will be utilized incessantly to recognize missing and inconsistencies data. The investigator will solve any problems promptly.

Statistical methods

All data will be analysed using SPSS version 21.0 (SPSS Inc Chicago, Illinois) with an intention-to-treat (ITT) principle, missing data will be imputed using multiple imputations. Statistical analyses will be done by a statistician who is blind to intervention allocations. Continuous variables will be described as means and standard deviation (SD) for normally and medians and interquartile range for abnormally distributed data. Comparisons between groups will performed with the Mann-Whitney U-test (analysis of variance) or Wilcoxon test, including cognitive load, adherence, duration of ICU delirium, length of ICU stay, length of stay in the hospital. Categorical variables will be expressed as frequencies and percentages. Chi-Square test or Fisher's exact test will used to examine between-group differences among incidence of delirium, baseline delirium risk factors and demographics.

Considering the correlation that results from repeated measurements on the same individual, repeated measures analysis of variance (RMANOVA) is used to analyze the multiple measurements variable which is gathered on everyday, such as CL,

adherence of ICU nurses. We will adjust for age, educational level, years of ICU experience, level of knowledge, since these factors were likely to be associated with adherence and CL. Bonferroni post hoc tests were used to assess within-group changes over time, as well as between-group differences during study intervention. Differences in incidence of delirium between groups are analyzed after adjusting for demographic variables using generalized estimation equation. All tests are two-tailed, and P - value < 0.05 is considered statistically significant.

Data monitoring

Supervisors adopt a safety role to monitor the quality and completeness of data in each centre. They will audit the original data and clarify any problems with the data in the collection (for example, insufficient enrollment or retention of participants, inadequate researchers, missing data). The supervisor have the right to access to terminate the trial in accordance with patients' safety. There is no interim analysis in this trial, and the study will go on until the target sample size is accomplished.

Harms

In this study, the intervention for patients in both groups are nursing activities that routinely used in daily care. Therefore, the present study will not pose an additional risk to patients. However, adverse events can occur even during normal care and result in patients' dysfunction, discomfortable signs, prolonged hospitalization or life-threatening events. Any adverse events (e.g. fall, pressure ulcer, unplanned extubation, bradycardia, tachycardia et al.) will be recorded by data collectors and reported to the Ethics Committee of Capital Medical University as soon as possible.

Strengths and Limitation

The strengths of the study include the following: Firstly, this study adopts a rigorous methodology to minimize hazard of potential bias. For instance, prior to the study, all

data collectors who unknown group allocation are trained to use the assessment tools to evaluate the variables proficiently and effectively. The operational definitions of variables involved in the study are predefined by reviewing literature and guidance to decrease information bias.Secondly, both doctors and nurses have poor adherence to guidelines in clinical practice, but researchers mostly discuss the reasons for poor adherence from the aspects of environmental factors, patient factors, disease factors and organizational management factors. This study will explore the role of CL in adherence to delirium interventions among ICU nurses, and provide theoretical and methodological basis for improving the adherence to delirium interventions. Fourthly, many methods are used to improve the adherence of healthcare staff, such as quality supervision, education, and increasing human resources. Our study developed a *AI-Antidelirium*, which aims to reduce extraneous CL of nurses and then improve the adherence of nurses to implement delirium interventions.

Several limitations should also be noted. One is that this study only carry out in two hospitals in a single region, which will limit the generalization of our results. The other limitation is the risk of contamination that nurses who involved in the study from the intervention and control ICUs in same hospital may communicate with each other during their non-working hours. Finally, our trial is not designed to evaluate long-term outcomes, such as cognitive load in ICU nurses. These issues in this trial should be addressed in future studies to further improve its efficiency and effectiveness.

References:

1. Sweller J. Cognitive load during problem solving： Effects on learning. *Cogn Sci*. 1988;12:257-285

2. Paas F, Renkl A, Sweller J. Cognitive Load Theory and Instructional Design：Recent Developments. *Educ Psychol (Lond)*. 2003;38(1):1

3. Paas FG, Van Merrienboer JJ, Adam JJ. Measurement of cognitive load in instructional research. *Percept Mot Skills*. 1994;79(1 Pt 2):419-30

4. Mohammadi M, Mazloumi A, Kazemi Z, Zeraati H. Evaluation of Mental Workload among ICU Ward's Nurses. *Health Promot Perspect*. 2015;5(4):280-7

5. Pawar S, Jacques T, Deshpande K, Pusapati R, Meguerdichian MJ. Evaluation of cognitive load and emotional states during multidisciplinary critical care simulation sessions. *BMJ Simul Technol Enhanc Learn*. 2018;4(2):87-91

6. Wheelock A, Suliman A, Wharton R et al. The Impact of Operating Room Distractions on Stress, Workload, and Teamwork. *Ann Surg*. 2015;261(6):1079-84

7. Sarsangi V, Khajevandi AA, Sarsangi F et al. Assessing Mental Workload and Factors that Influence it among Nursing Staff in Emergency Departments. *J Mazandaran Univ Med Sci*. 2015;25(126):155-158

8. Starmer AJ, O'Toole JK, Rosenbluth G et al. Development, implementation, and dissemination of the I-PASS handoff curriculum: A multisite educational intervention to improve patient handoffs. *Acad Med*. 2014;89(6):876-84

9. Song HS, Pusic M, Nick MW et al. The cognitive impact of interactive design features for learning complex materials in medical education. *Comput Educ*. 2014;71:198-205

10. Ceballos-Vasquez P, Rolo-Gonzalez G, Hernandez-Fernaud E et al. Psychosocial factors and mental work load: a reality perceived by nurses in intensive care units. *Rev Lat Am Enfermagem*. 2015;23(2):315-22

11. Wu P, Nam MY, Choi J et al. Supporting Emergency Medical Care Teams with an Integrated Status Display Providing Real-Time Access to Medical Best Practices, Workflow Tracking, and Patient Data. *J Med Syst*. 2017;41(12):186

12. Klein KP, Zaal IJ, Spitoni C et al. The attributable mortality of delirium in critically ill patients:

prospective cohort study. *BMJ*. 2014;349:g6652

13. Ely EW, Shintani A, Truman B et al. Delirium as a predictor of mortality in mechanically ventilated patients in the intensive care unit. *JAMA*. 2004;291(14):1753-62
14. Elliott SR. ICU delirium: a survey into nursing and medical staff knowledge of current practices and perceived barriers towards ICU delirium in the intensive care unit. *Intensive Crit Care Nurs*. 2014;30(6):333-8
15. Noriega FJ, Vidan MT, Sanchez E et al. Incidence and impact of delirium on clinical and functional outcomes in older patients hospitalized for acute cardiac diseases. *Am Heart J*. 2015;170(5):938-44
16. Hshieh TT, Yue J, Oh E et al. Effectiveness of multicomponent nonpharmacological delirium interventions: a meta-analysis. *JAMA Intern Med*. 2015;175(4):512-20
17. Oh ES, Fong TG, Hshieh TT, Inouye SK. Delirium in Older Persons: Advances in Diagnosis and Treatment. *JAMA*. 2017;318(12):1161-1174
18. Barr J. Clinical Practice Guidelines for the Management of Pain, Agitation, and Delirium in Adult Patients in the Intensive Care Unit. *Crit Care Med*. 2013;41(1):263-306
19. Brummel NE, Bell SP, Girard TD et al. Frailty and Subsequent Disability and Mortality among Patients with Critical Illness. *Am J Respir Crit Care Med*. 2017;196(1):64-72
20. Devlin JW, Skrobik Y, Gelinas C et al. Clinical Practice Guidelines for the Prevention and Management of Pain, Agitation/Sedation, Delirium, Immobility, and Sleep Disruption in Adult Patients in the ICU. *Crit Care Med*. 2018;46(9):e825-e873
21. Miller MA, Govindan S, Watson SR, Hyzy RC, Iwashyna TJ. ABCDE, but in that order? A cross-sectional survey of Michigan intensive care unit sedation, delirium, and early mobility practices. *Ann Am Thorac Soc*. 2015;12(7):1066-71
22. Morandi A, Piva S, Ely EW et al. Worldwide Survey of the "Assessing Pain, Both Spontaneous Awakening and Breathing Trials, Choice of Drugs, Delirium Monitoring/Management, Early Exercise/Mobility, and Family Empowerment" (ABCDEF) Bundle. *Crit Care Med*. 2017;45(11):e1111-e1122
23. Balas MC, Burke WJ, Gannon D et al. Implementing the awakening and breathing coordination, delirium monitoring/management, and early exercise/mobility bundle into everyday care: opportunities, challenges, and lessons learned for implementing the ICU Pain, Agitation, and Delirium Guidelines. *Crit Care Med*. 2013;41(9 Suppl 1):S116-27

24. Saczynski JS, Kosar CM, Xu G et al. A tale of two methods: chart and interview methods for identifying delirium. *J Am Geriatr Soc*. 2014;62(3):518-24
25. Alhaidari AA, Allen-Narker RA. An evolving approach to delirium: A mixed-methods process evaluation of a hospital-wide delirium program in New Zealand. *Australas J Ageing*. 2017;36(2):E20-E26
26. Hosie A, Lobb E, Agar M, Davidson PM, Phillips J. Identifying the barriers and enablers to palliative care nurses' recognition and assessment of delirium symptoms: a qualitative study. *J Pain Symptom Manage*. 2014;48(5):815-30
27. Xing J, Sun Y, Jie Y, Yuan Z, Liu W. Perceptions, attitudes, and current practices regards delirium in China: A survey of 917 critical care nurses and physicians in China. *Medicine (Baltimore)*. 2017;96(39):e8028
28. Vogels J, Demberg V, Kray J. The Index of Cognitive Activity as a Measure of Cognitive Processing Load in Dual Task Settings. *Front Psychol*. 2018;9:2276
29. Kirschner PA, Sweller J, Kirschner F, Zambrano RJ. From Cognitive Load Theory to Collaborative Cognitive Load Theory. *Int J Comput Support Collab Learn*. 2018;13(2):213-233
30. Rouleau G, Gagnon MP, Cote J et al. Impact of Information and Communication Technologies on Nursing Care: Results of an Overview of Systematic Reviews. *J Med Internet Res*. 2017;19(4):e122
31. Wang PF, Shen LQ, Zhang HJ, Li BH, Ji H. A Nursing Pain Assessment and Record Information System: Design and Application in the Oncology Department. *Comput Inform Nurs*. 2017;35(12):647-652
32. Gu D, Li J, Li X, Liang C. Visualizing the knowledge structure and evolution of big data research in healthcare informatics. *Int J Med Inform*. 2017;98:22-32
33. Baad M, Lu ZF, Reiser I, Paushter D. Clinical Significance of US Artifacts. *Radiographics*. 2017;37(5):1408-1423
34. Gebodh N, Esmaeilpour Z, Adair D et al. Inherent physiological artifacts in EEG during tDCS. *Neuroimage*. 2019;185:408-424
35. Lyell D, Magrabi F, Coiera E. The Effect of Cognitive Load and Task Complexity on Automation Bias in Electronic Prescribing. *Hum Factors*. 2018;60(7):1008-1021
36. Pickering BW, Herasevich V, Ahmed A, Gajic O. Novel Representation of Clinical Information in

the ICU: Developing User Interfaces which Reduce Information Overload. *Appl Clin Inform*. 2010;1(2):116-31

37. Wu P, Nam MY, Choi J et al. Supporting Emergency Medical Care Teams with an Integrated Status Display Providing Real-Time Access to Medical Best Practices, Workflow Tracking, and Patient Data. *J Med Syst*. 2017;41(12):186
38. Dal Sasso GM, Barra DC. Cognitive Workload of Computerized Nursing Process in Intensive Care Units. *Comput Inform Nurs*. 2015;33(8):339-45; quiz E1
39. Chan AW, Tetzlaff JM, Gotzsche PC et al. SPIRIT 2013 explanation and elaboration: guidance for protocols of clinical trials. *BMJ*. 2013;346:e7586
40. Fan H, Ji M, Huang J et al. Development and validation of a dynamic delirium prediction rule in patients admitted to the Intensive Care Units (DYNAMIC-ICU): A prospective cohort study. *Int J Nurs Stud*. 2019;93:64-73
41. Leppink J, Paas F, Van der Vleuten CP, Van Gog T, Van Merrienboer JJ. Development of an instrument for measuring different types of cognitive load. *Behav Res Methods*. 2013;45(4):1058-72
42. Trogrlic Z, van der Jagt M, Lingsma H et al. Improved Guideline Adherence and Reduced Brain Dysfunction After a Multicenter Multifaceted Implementation of ICU Delirium Guidelines in 3,930 Patients. *Crit Care Med*. 2019;47(3):419-427
43. Dykes PC, Carroll DL, Hurley A et al. Fall prevention in acute care hospitals: a randomized trial. *JAMA*. 2010;304(17):1912-8
44. Xing J, Sun Y, Jie Y, Yuan Z, Liu W. Perceptions, attitudes, and current practices regards delirium in China: A survey of 917 critical care nurses and physicians in China. *Medicine (Baltimore)*. 2017;96(39):e8028
