# Supplementary material for: Impact of Clinical Decision Support System Assisted prevention and management for Delirium on guideline adherence and cognitive load among Intensive Care Unit nurses (CDSSD-ICU): Protocol of a multicentre, cluster randomized trial
Source: PLoS One. 2023 Nov 28;18(11):e0293950. doi: 10.1371/journal.pone.0293950 (PMC10684021; doi:10.1371/journal.pone.0293950)
Supplement: S6 File — (PDF) [file pone.0293950.s006.pdf]

S6 File. Research project submitted to the ethics committee (Chinese).

题目：认知负荷在影响ICU护士谵妄护理依从性中的作用及机制

博士生:张山

指导教师:吴瑛教授

单位:首都医科大学

总结

护士认知负荷高是重症监护室的一个普遍问题,这与护士的活动表现不佳有关，也与患者预后不佳有关。目前尚不清楚人工智能辅助谵妄预防和管理(Artificial Intelligence Assisted Prevention and Management for Delirium，AI-AntiDelirium)是否能降低ICU护士的认知负荷，提高谵妄干预的依从性。我们旨在评估AI-AntiDelirium对ICU护士认知负荷和谵妄干预依从性的有效性，以及ICU谵妄的影响。这是一项多中心、前瞻性、聚类随机对照临床试验，比较AI-AntiDelirium和PADIS指南对ICU护士的影响。两家医院共6个ICU将按1:1的比例随机接受AI-AntiDelirium(干预组)或PADIS指南(对照组)，目标样本量为78名ICU护士。主要终点将是谵妄干预的依从性。次要终点将是认知负荷，由测量不同类型认知负荷的仪器(measuring different types of cognitive load，MDT-认知负荷)测量。患者的终点包括ICU谵妄的发生率和持续时间、ICU和住院时间、ICU和住院死亡率。结果评估将由无视分组分配的调查人员进行。采用重复测量方差分析(RMANOVA)检测各组依从性和认知负荷的差异。将执行广义估计方程来测试患者预后的差异。

研究背景

认知负荷是指一个人处理认知活动所需的认知资源总量[1]，由三种不同类型的认知负荷组成:内在负荷、外在负荷和关联负荷[2,3]。高认知负荷，即超过一个人的工作记忆资源容量，已被确定为在提供重症监护时最重要的问题之一。它会对护士和病人产生负面影响[4]，导致护士易怒、记忆障碍和精神疲劳[6,7]，不利于护理措施的实施，降低获取知识的学习能力[8,9]，还会导致病人预后不佳并危及病

人安全[6]。护士执行的活动越复杂，对认知负荷的要求就越高，这将阻碍护士坚持实施护理活动[10,11]。

重症监护室(intensive care units，ICU)护理的特点是工作量大、活动复杂、决策复杂。一个例子是ICU谵妄预防和管理干预所需的复杂护理。ICU谵妄是ICU患者的常见并发症，发生率高达70%～87%[12,13]，与住院时间延长(LOS)和死亡率增加相关[13-15]。因此，《ICU成人患者疼痛、躁动/镇静、谵妄、不动和睡眠中断的预防和管理临床实践指南》(PADIS指南)推荐使用ABCDEF包作为预防和管理ICU谵妄的一种方法，其重点是消除ICU谵妄的危险因素[18-20]。然而，在常规临床护理中，ABCDEF组合的依从性并不理想[21,22]。既往研究表明，护理记录工作量大[23]、评估工具算法复杂[24,25]、难以通过多渠道收集和记忆众多危险因素[26]、缺乏对ICU谵妄的认识等障碍，可能会阻碍集束化措施的依从实施[27]。在复杂的活动中工作导致接收和处理信息的速度降低，工作记忆能力下降，需要更高的认知负荷[28,29]。

考虑到谵妄护理中高认知负荷的不良后果，为ICU护士提供一种降低ICU谵妄护理中认知负荷的工具是很重要的。随着信息技术的飞速发展，临床决策支持系统(clinical decision support system，CDSS)已被世界上大多数医院广泛用于疾病评估、管理和记录[11,30-32]。CDSS在临床护理中有重要作用，在认知心理学中也有广泛应用。它可以对患者信息进行收集、整理、分类和建立逻辑关系，并利用预警、信息反馈为疾病诊断、治疗和护理活动提供决策支持[35,36]。多项研究表明，CDSS可以帮助医护人员回忆较少的临床信息，从而显著降低认知负荷，提高实施护理干预的依从性[37,38]。

为此，我们开发了一套人工智能辅助谵妄预防和管理系统(AI-AntiDelirium)，包括ABCDEF集束化干预、高危因素和ICU谵妄评估工具。本研究旨在评估AI-AntiDelirium对ICU护士依从性和认知负荷的影响。

方法

该方案是根据标准方案项目:干预性试验建议(the Standard Protocol Items:

Recommendations for Interventional Trial，SPIRIT)制定的[39]。

研究设计

AI-AntiDelirium 提高 ICU 护士谵妄干预依从性的研究是一项多中心、整群随机对照试验(RCT)，选取了两家三级医院 ICU 谵妄发生率较高的 6 个 ICU，分别代表 3 种 ICU 类型(2 个外科 ICU [SICU]、2 个呼吸 ICU [RICU]和 2 个心脏 ICU [CICU])以及样本的多样性。A 医院是一所拥有 1000 张床位的大学附属教学医院，其中 3 个参与 ICU 拥有 42 张成人 ICU 床位。B 医院有床位 730 张，3 个 ICU 包含 46 张，年入院率约 850 人。全面实施医院信息化建设。该研究将于 2022 年至 2023 年期间进行。图 1 显示了研究的设计。

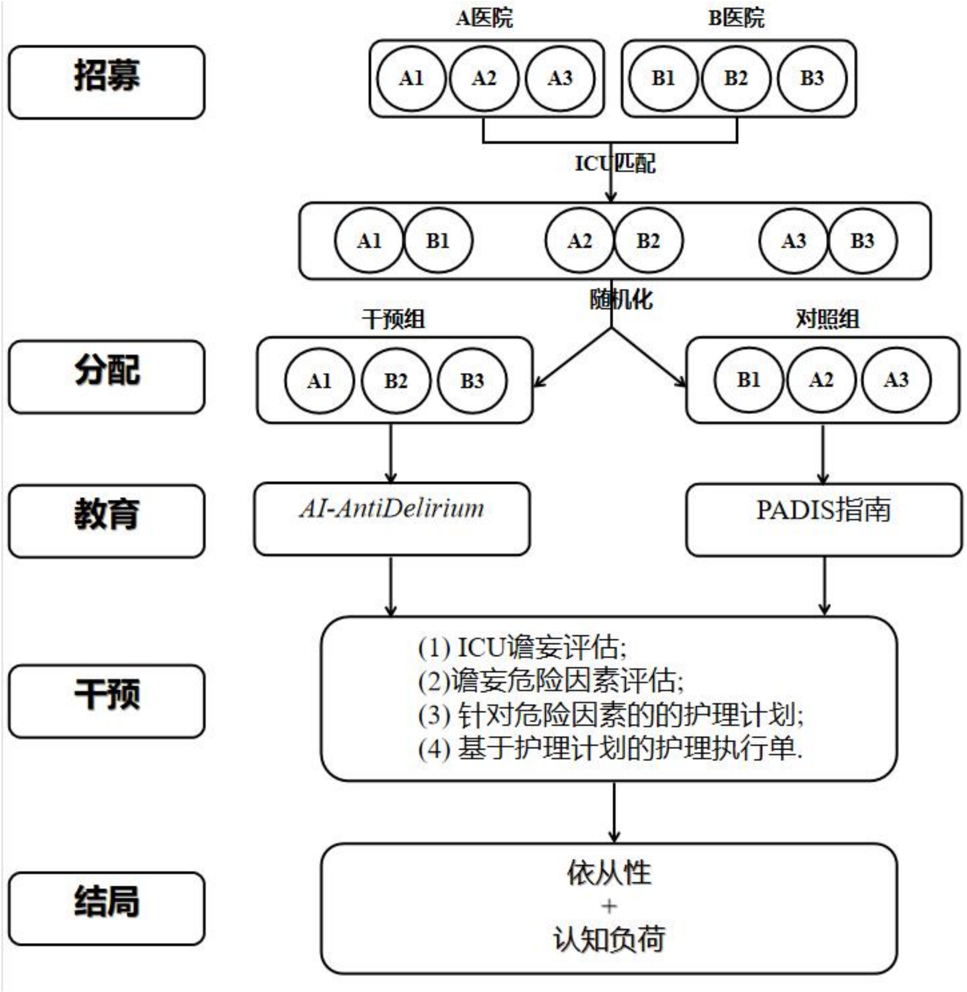

图 1 研究设计

AI-AntiDelirium, Artificial Intelligence Assisted Prevention and Management for Delirium;
PADIS guideline, Clinical Practice Guidelines for the Prevention and Management of Pain, Agitation/Sedation, Delirium, Immobility, and Sleep Disruption in Adult Patients in the ICU;

研究对象

全部招募在纳入 ICU 工作的注册护士(RN)。护士有资格参加这项研究，纳入标准:(1)至少有 1 年的重症监护经验;(2)在本单位全职工作;(3)同意参加本研究。在研究期间，在医院外参加学习项目或因各种原因休假的护士被排除在外。

干预组

在研究之前，研究人员提供了一个教育计划，包括有关 ICU 谵妄风险因素的知识，评估工具(重症监护室混淆评估方法[CAM-ICU]，重症监护谵妄筛查检查表[ICDSC])和 ABCDEF 集束化干预措施。除此之外，合格的 ICU 护士接受了如何操作 AI-AntiDelirium 的培训。我们开发了 AI-AntiDelirium，包括四个主要模块:ICU 谵妄评估模块(图 1A)、危险因素评估模块(图 1B)、护理计划模块(图 1C)和护理执行单模块(图 1D)。

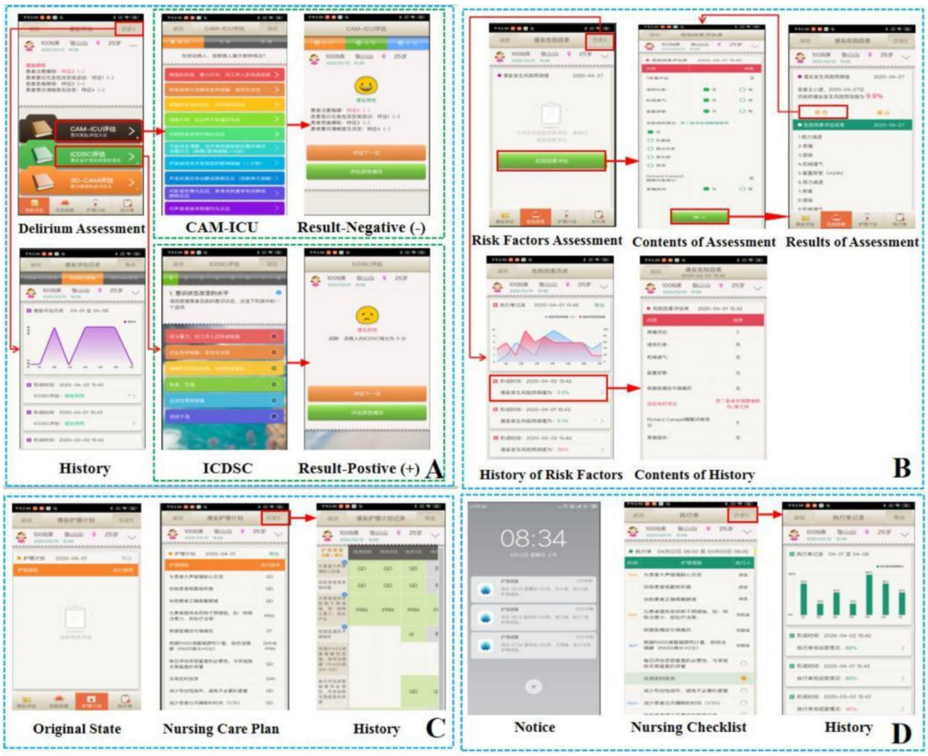

图 1. AI-AntiDelirium 的应用模块

A: ICU 谵妄评估工具及结果; B:风险因素评估和风险值预测; C:护理计划;D:护理执行单

干预组 ICU 护士基于 AI-AntiDelirium 进行 ICU 谵妄护理。每天，护士在

AI-AntiDelirium 辅助下对 ICU 患者进行评估，AI-AntiDelirium 可识别 ICU 谵妄，自动提示危险因素，量身定制循证 ICU 谵妄预防或管理策略。在研究过程中，接受 ICU 谵妄训练的护士在个人数字助理(PDA)上点击 AI-AntiDelirium 图标进入系统。

A.报名:点击报名图标→输入用户名→输入密码→选择医院和科室→提交;

B.登录:输入用户名和密码→登录。

C.添加患者:点击“+”按钮→输入病案号。→输入患者姓名→输入患者性别→输入患者年龄→输入患者床号→输入患者入院时间。

D.诊断 ICU 谵妄:点击谵妄评估按钮→选择谵妄评估工具(CAM-ICU 或 ICDSC)→根据提示逐项完成→AI-AntiDelirium 会自动显示患者是否有谵妄。

E.识别 ICU 患者谵妄的危险因素:点击“危险因素评估”按钮→完成一份简要的危险因素评估评估清单，包括易感因素(如听力障碍、视力障碍)、疾病相关因素(如感染、疼痛)、医源性因素和环境因素(如机械通气→AI-AntiDelirium 将自动报告患者的个性化危险因素→AI-AntiDelirium 将根据 ICU 动态谵妄预测规则自动显示发生谵妄的风险预测值 [40])。

F.护理计划确认:点击“护理计划”按钮→AI-AntiDelirium 会根据危险因素评估结果自动显示单个 ICU 谵妄预防或管理护理方案→ICU 护士检查并确认对患者的所有干预措施都是适当可行的。

G.实施针对个别风险因素的干预措施:点击“护理执行单”按钮→AI-AntiDelirium 会根据护理计划的结果自动自动显示每项干预的具体执行时间→护士在整个班次中实施个别干预并记录患者未接受这些干预的原因→护士实施后点击措施→AI-AntiDelirium 自动记录护士的姓名和执行时间。

对照组

研究开始前，由研究人员进行 ICU 谵妄相关的教育项目，内容与干预组相同。此外，护士还接受了使用 PADIS 指南的培训。在研究过程中，护士将根据 PADIS 指南进行 ICU 谵妄护理，包括 ICU 谵妄危险因素、评估工具(CAM-ICU、

ICDSC)、ABCDEF 捆绑干预。

A.诊断 ICU 谵妄:护士选择一种仪器(CAM-ICU 或 ICDSC)评估 ICU 谵妄发生情况，并逐一完成。护士手动记录每个项目的分配分数，并计算出最终的总分，然后根据评估工具的规则判断患者是否出现谵妄。

B.确定患者 ICU 谵妄危险因素:ICU 护士填写简短的谵妄危险因素评估(内容与干预组相同)。护士根据危险因素评估结果自行判断患者当前的危险因素，并根据 ICU 谵妄动态预测规则手动计算患者当前发生 ICU 谵妄的风险。

C.实施个体化、针对危险因素的干预措施:护士根据患者具体危险因素的结果，从 PADIS 指南中提取护理干预措施，并记录在护理记录中，实施相应的干预措施子集。

结局指标

结局指标评估将由不参与患者临床护理的训练有素的研究人员实施。主要指标为干预依从性，定义为护士每天实施 ICU 谵妄及危险因素评估、谵妄预防和管理干预的依从性。干预工作人员将每天记录不遵守的原因。

A.谵妄评估依从性：计划护士每天对患者至少进行两次谵妄评估，每位护士每天理论谵妄评估的次数为 2×每位护士管理纳入患者数，则护士谵妄评估依从性 = 护士每天实际评估谵妄次数/理论评估次数；

B.谵妄危险因素评估依从性：计划护士每天对患者至少进行一次谵妄危险因素评估，目前危险因素评估单主要针对谵妄的 10 个危险因素进行评估，每位护士每天理论谵妄危险因素评估的数目为 10×每位护士管理纳入的患者数，则护士谵妄危险因素评估依从性 = 护士每天实际评估谵妄危险因素数目/护士理论评估数目；

C.谵妄预防和干预措施依从性：护士实施谵妄预防和干预措施的依从性 = 护士实际完成谵妄护理措施条目数/护士理论应该完成措施条目数；智能组护士理论应该完成谵妄护理措施条目数根据 AI-AntiDelirium 自动制定的个性化谵妄预防或干预措施来明确，实际完成谵妄护理措施条目数则根据 AI-AntiDelirium 中

记录的护士实际执行情况来确定；非智能组护士理论应该完成护理措施条目数则由研究人员将护士实施谵妄危险因素评估的结果输入到 AI-AntiDelirium，基于该系统提供的谵妄预防或干预措施来明确非智能组理论应该完成护理措施条目数，实际完成谵妄护理措施条目数则根据护理记录以及与护士核对来明确。

次要结局

护士的认知负荷，是指人在处理认知任务时，所需认知资源的总量；应用中文版 MDT-CL 量表测量，该工具有 10 个项目，已成功用于区分不同类型的认知负荷[41]。每项得分从 0 到 10 分，得分越高，认知负荷越高。中文版 MDT-认知负荷的 Cronbach 's α为 0.818，测量内在认知负荷(第 1、2、3 项)的 Cronbach 's α值为 0.879，测量外在认知负荷(第 4、5、6 项)的 cronbach 's α值为 0.878，测量相关认知负荷(第 7、8、9 和 10 项)的 cronbach 's α值为 0.946。

研究对象的时间表

纳入和数据收集于 2022 年 11 月开始。纳入将继续进行，直到目标人群(78 名护士)招募完毕，预计将于 2023 年 6 月结束。之后，数据分析将用于出版物。

样本量计算

该整群随机对照研究的目的是与 PADIS 指南相比，通过使用 AI-AntiDelirium 来提高 ICU 护士对谵妄干预的依从性。我们预计 AI-Antidelirium 组谵妄干预的依从性为 80%，PADIS 指南组为 50%[42]，提示 AI-Antidelirium 对提高 ICU 护士谵妄干预的依从性具有临床意义。样本量计算显示，每个集群中 12 名护士将提供 80%的功率，双侧显著性水平(α)为 0.05，集群内(单位内)相关性为 0.002[43]。另外，考虑到可能的 10%的退出率，我们计划在每个 ICU 招募 13 名护士，最终样本量为 78(13*6)名护士。

纳入

所有的纳入都将由训练有素的研究人员进行，他们不参与干预，也不了解护士的小组任务。研究人员将根据纳入和排除标准对护士进行日常筛查。将获得所有合格护士的书面知情同意。为了留住更多的护士，研究人员将解释如果这项研

究成功实施对护士的好处。

随机

通过整群随机分组降低组间污染的风险，ICU 将随机 1:1 接受 AI-AntiDelirium 或 PADIS 指南。我们计划在两家医院招募 6 名 ICU，并匹配 ICU 类型(考虑不同类型 ICU 护理流程、工作量、患者病情严重程度等影响护士依从性的因素)。分配序列基于计算机生成的随机数，由独立于数据分析且不参与数据收集的统计学家执行。为了保证分配的隐蔽性，统计人员将每个 ICU 的分配代码告知研究协调员。然后，研究协调员会通知 ICU 护士他们被分配到哪一组。招募所有符合条件的护士，直到样本量足够为止。

盲法

将招募参与者的研究人员不知道随机化列表，患者也不知道他们的分配。但是实施策略的护士不可能因为干预的性质而盲目分配。基线数据和终点措施将由数据收集者或结果评估者收集，他们在干预中没有参与，对分配一无所知。

数据收集

如表 1 所示，在研究开始前，研究人员将对数据收集人员进行统一培训。所有的研究数据都是匿名和保密的。在参与研究前，将获得所有 ICU 护士的知情同意。然后收集 ICU 护士的基本人口学数据，包括:年龄、性别、婚姻状况、教育背景、工作科室、ICU 工作年限、职称、基线认知负荷、知识水平。知识水平是指护士通过学习或临床实践获得的对 ICU 谵妄评估工具、危险因素、预防和干预的知识，并使用 ICU 谵妄知识问卷进行测量。新编制了一份 20 项选择题知识问卷，内容效度为 0.96，问卷整体 cronbach’s α值为 0.814[44]。得分越高，表示对 ICU 谵妄的了解程度越高。在研究期间，每天轮班结束时记录 ICU 护士的认知负荷和对干预措施的依从性。数据存储在由数据管理器驱动的电子文件中。

表 1 ICU 护士依从谵妄干预改善方案时间表

时间点 / 评估内容	招募	基线评估	每日评估	结局评估

**招募**			
筛选	×		
知情同意	×		
**基线评估**			
年龄		×	
性别		×	
婚姻状况		×	
教育水平		×	
种族		×	
科室		×	
ICU 工作年限		×	
职称		×	
知识水平		×	
**分配**		×	
**干预**			
AI-AntiDelirium			×
Step1: ICU 谵妄评估			×
Step2: 危险因素评估			×
Step3: 护理计划			×
Step4: 护理执行单			×
**纸质版 PADIS 指南**			×
Step1: ICU 谵妄评估			×
Step2: 危险因素评估			×
Step3: 护理计划			×
Step4: 护理执行单			×

**结局**			
ICU 谵妄评估依从性		×	×
危险因素评估依从性		×	×
谵妄预防及干预措施依从性		×	×
认知负荷	×	×	×

数据管理

所有数据将收集在打印的、预先编码的表格上，该表格将两次输入电子数据库，并进行广泛的错误检查和数据完整性。参与这项研究的重症监护室只能访问他们自己的数据。为了提高数据质量，我们将进行以下流程:(1)在研究开始前，所有调查人员和数据收集人员都将接受培训，掌握数据收集的流程;(2)所有参与的护士将接受工具应用培训，但不同组护士将参加不同的教育环节，干预组护士将学习如何使用 AI-AntiDelirium，对照组护士将学习如何使用 PADIS 指南;(3)不断对数据进行清理和审核，识别缺失和不一致的数据。调查人员将及时解决任何问题。

统计方法

所有数据将使用 SPSS 21.0 版本(SPSS Inc 芝加哥，伊利诺伊州)分析，并遵循意向治疗(ITT)原则，缺失数据将使用多重插补进行估算。统计分析将由一名统计学家完成，他对干预分配一无所知。连续变量将被描述为正态和中位数的均值和标准偏差(SD)，以及异常分布数据的四分位极差。两组比较采用 Mann-Whitney u 检验(方差分析)或 Wilcoxon 检验，包括认知负荷、依从性、ICU 谵妄持续时间、ICU 住院时间、住院时间。分类变量将表示为频率和百分比。卡方检验或 Fisher 精确检验将用于检查谵妄发生率、基线谵妄危险因素和人口统计学之间的组间差异。

考虑到对同一个体的重复测量产生的相关性，采用重复测量方差分析(RMANOVA)对每天收集的多个测量变量进行分析，如认知负荷、ICU 护士的依

从性等。我们将根据年龄、教育水平、ICU 经验年数、知识水平进行调整，因为这些因素可能与依从性和认知负荷有关。Bonferroni 事后测试用于评估组内随时间的变化，以及研究干预期间组间差异。采用广义估计方程对人口学变量进行调整后，分析各组谵妄发生率的差异。所有检验均为双侧检验，P 值< 0.05被认为有统计学意义。

数据监控

监督员扮演安全角色，监测每个中心数据的质量和完整性。他们将审核原始数据，并澄清收集数据中的任何问题(例如，参与者注册或保留不足，研究人员不足，数据缺失)。监督者有权根据患者的安全要求终止试验。本试验没有中期分析，研究将继续进行，直到完成目标样本量为止。

危害

在本研究中，两组患者的干预均为日常护理中常规使用的护理活动。因此，本研究不会给患者带来额外的风险。然而，即使在正常护理期间，不良事件也可能发生，导致患者功能障碍、不适体征、延长住院时间或危及生命的事件。任何不良事件(如跌倒、压性溃疡、意外拔管、心动过缓、心动过速等)将由数据收集人员记录，并尽快上报首都医科大学伦理委员会。

优势与局限性

本研究的优势包括以下几个方面:首先，本研究采用了严格的方法，最大限度地减少潜在偏倚的风险。例如，在研究之前，所有未知组分配的数据收集者都接受了使用评估工具熟练有效地评估变量的培训。本研究中涉及的变量的操作定义是通过回顾文献和指南来预先定义的，以减少信息偏差。其次，在临床实践中，医生和护士对指南的依从性都很差，但研究人员多从环境因素、患者因素、疾病因素和组织管理因素等方面讨论依从性差的原因。本研究旨在探讨认知负荷在 ICU 护士谵妄干预依从性中的作用，为提高 ICU 护士谵妄干预依从性提供理论和方法依据。第四，采用多种方法提高医护人员的依从性，如质量监督、教育和增加人力资源。我们的研究开发了一种 AI-Antidelirium，旨在减少护士的外来认

知负荷，从而提高护士实施谵妄干预的依从性。

局限性如下：一是本研究仅在一个地区的两家医院进行，这限制了我们结果的泛化。另一个限制是，参与研究的护士在非工作时间可能会在同一家医院的干预 ICU 和对照 ICU 相互交流，存在污染风险。最后，我们的试验并不是为了评估长期结果，比如 ICU 护士的认知负荷。本试验的这些问题应在今后的研究中加以解决，以进一步提高其效率和效果。

References:

1. Sweller J. Cognitive load during problem solving：Effects on learning. Cogn Sci. 1988;12:257-285
2. Paas F, Renkl A, Sweller J. Cognitive Load Theory and Instructional Design：Recent Developments. Educ Psychol (Lond). 2003;38(1):1
3. Paas FG, Van Merrienboer JJ, Adam JJ. Measurement of cognitive load in instructional research. Percept Mot Skills. 1994;79(1 Pt 2):419-30
4. Mohammadi M, Mazloumi A, Kazemi Z, Zeraati H. Evaluation of Mental Workload among ICU Ward's Nurses. Health Promot Perspect. 2015;5(4):280-7
5. Pawar S, Jacques T, Deshpande K, Pusapati R, Meguerdichian MJ. Evaluation of cognitive load and emotional states during multidisciplinary critical care simulation sessions. BMJ Simul Technol Enhanc Learn. 2018;4(2):87-91
6. Wheelock A, Suliman A, Wharton R et al. The Impact of Operating Room Distractions on Stress, Workload, and Teamwork. Ann Surg. 2015;261(6):1079-84
7. Sarsangi V, Khajevandi AA, Sarsangi F et al. Assessing Mental Workload and Factors that Influence it among Nursing Staff in Emergency Departments. J Mazandaran Univ Med Sci. 2015;25(126):155-158

8. Starmer AJ, O'Toole JK, Rosenbluth G et al. Development, implementation, and dissemination of the I-PASS handoff curriculum: A multisite educational intervention to improve patient handoffs. Acad Med. 2014;89(6):876-84

9. Song HS, Pusic M, Nick MW et al. The cognitive impact of interactive design features for learning complex materials in medical education. Comput Educ. 2014;71:198-205

10. Ceballos-Vasquez P, Rolo-Gonzalez G, Hernandez-Fernaud E et al. Psychosocial factors and mental work load: a reality perceived by nurses in intensive care units. Rev Lat Am Enfermagem. 2015;23(2):315-22

11. Wu P, Nam MY, Choi J et al. Supporting Emergency Medical Care Teams with an Integrated Status Display Providing Real-Time Access to Medical Best Practices, Workflow Tracking, and Patient Data. J Med Syst. 2017;41(12):186

12. Klein KP, Zaal IJ, Spitoni C et al. The attributable mortality of delirium in critically ill patients: prospective cohort study. BMJ. 2014;349:g6652

13. Ely EW, Shintani A, Truman B et al. Delirium as a predictor of mortality in mechanically ventilated patients in the intensive care unit. JAMA. 2004;291(14):1753-62

14. Elliott SR. ICU delirium: a survey into nursing and medical staff knowledge of current practices and perceived barriers towards ICU delirium in the intensive care unit. Intensive Crit Care Nurs. 2014;30(6):333-8

15. Noriega FJ, Vidan MT, Sanchez E et al. Incidence and impact of delirium on clinical and functional outcomes in older patients hospitalized for acute cardiac diseases. Am Heart J. 2015;170(5):938-44

16. Hshieh TT, Yue J, Oh E et al. Effectiveness of multicomponent nonpharmacological delirium interventions: a meta-analysis. JAMA Intern Med. 2015;175(4):512-20

17. Oh ES, Fong TG, Hshieh TT, Inouye SK. Delirium in Older Persons: Advances in Diagnosis and Treatment. JAMA. 2017;318(12):1161-1174

18. Barr J. Clinical Practice Guidelines for the Management of Pain, Agitation, and Delirium in Adult Patients in the Intensive Care Unit. Crit Care Med. 2013;41(1):263-306
19. Brummel NE, Bell SP, Girard TD et al. Frailty and Subsequent Disability and Mortality among Patients with Critical Illness. Am J Respir Crit Care Med. 2017;196(1):64-72
20. Devlin JW, Skrobik Y, Gelinas C et al. Clinical Practice Guidelines for the Prevention and Management of Pain, Agitation/Sedation, Delirium, Immobility, and Sleep Disruption in Adult Patients in the ICU. Crit Care Med. 2018;46(9):e825-e873
21. Miller MA, Govindan S, Watson SR, Hyzy RC, Iwashyna TJ. ABCDE, but in that order? A cross-sectional survey of Michigan intensive care unit sedation, delirium, and early mobility practices. Ann Am Thorac Soc. 2015;12(7):1066-71
22. Morandi A, Piva S, Ely EW et al. Worldwide Survey of the "Assessing Pain, Both Spontaneous Awakening and Breathing Trials, Choice of Drugs, Delirium Monitoring/Management, Early Exercise/Mobility, and Family Empowerment" (ABCDEF) Bundle. Crit Care Med. 2017;45(11):e1111-e1122
23. Balas MC, Burke WJ, Gannon D et al. Implementing the awakening and breathing coordination, delirium monitoring/management, and early exercise/mobility bundle into everyday care: opportunities, challenges, and lessons learned for implementing the ICU Pain, Agitation, and Delirium Guidelines. Crit Care Med. 2013;41(9 Suppl 1):S116-27
24. Saczynski JS, Kosar CM, Xu G et al. A tale of two methods: chart and interview methods for identifying delirium. J Am Geriatr Soc. 2014;62(3):518-24
25. Alhaidari AA, Allen-Narker RA. An evolving approach to delirium: A mixed-methods process evaluation of a hospital-wide delirium program in New Zealand. Australas J Ageing. 2017;36(2):E20-E26
26. Hosie A, Lobb E, Agar M, Davidson PM, Phillips J. Identifying the barriers and enablers to palliative care nurses' recognition and assessment of delirium symptoms: a

qualitative study. J Pain Symptom Manage. 2014;48(5):815-30

27. Xing J, Sun Y, Jie Y, Yuan Z, Liu W. Perceptions, attitudes, and current practices regards delirium in China: A survey of 917 critical care nurses and physicians in China. Medicine (Baltimore). 2017;96(39):e8028

28. Vogels J, Demberg V, Kray J. The Index of Cognitive Activity as a Measure of Cognitive Processing Load in Dual Task Settings. Front Psychol. 2018;9:2276

29. Kirschner PA, Sweller J, Kirschner F, Zambrano RJ. From Cognitive Load Theory to Collaborative Cognitive Load Theory. Int J Comput Support Collab Learn. 2018;13(2):213-233

30. Rouleau G, Gagnon MP, Cote J et al. Impact of Information and Communication Technologies on Nursing Care: Results of an Overview of Systematic Reviews. J Med Internet Res. 2017;19(4):e122

31. Wang PF, Shen LQ, Zhang HJ, Li BH, Ji H. A Nursing Pain Assessment and Record Information System: Design and Application in the Oncology Department. Comput Inform Nurs. 2017;35(12):647-652

32. Gu D, Li J, Li X, Liang C. Visualizing the knowledge structure and evolution of big data research in healthcare informatics. Int J Med Inform. 2017;98:22-32

33. Baad M, Lu ZF, Reiser I, Paushter D. Clinical Significance of US Artifacts. Radiographics. 2017;37(5):1408-1423

34. Gebodh N, Esmaeilpour Z, Adair D et al. Inherent physiological artifacts in EEG during tDCS. Neuroimage. 2019;185:408-424

35. Lyell D, Magrabi F, Coiera E. The Effect of Cognitive Load and Task Complexity on Automation Bias in Electronic Prescribing. Hum Factors. 2018;60(7):1008-1021

36. Pickering BW, Herasevich V, Ahmed A, Gajic O. Novel Representation of Clinical Information in the ICU: Developing User Interfaces which Reduce Information Overload. Appl Clin Inform. 2010;1(2):116-31

37. Wu P, Nam MY, Choi J et al. Supporting Emergency Medical Care Teams with an Integrated Status Display Providing Real-Time Access to Medical Best Practices, Workflow Tracking, and Patient Data. J Med Syst. 2017;41(12):186

38. Dal Sasso GM, Barra DC. Cognitive Workload of Computerized Nursing Process in Intensive Care Units. Comput Inform Nurs. 2015;33(8):339-45; quiz E1

39. Chan AW, Tetzlaff JM, Gotzsche PC et al. SPIRIT 2013 explanation and elaboration: guidance for protocols of clinical trials. BMJ. 2013;346:e7586

40. Fan H, Ji M, Huang J et al. Development and validation of a dynamic delirium prediction rule in patients admitted to the Intensive Care Units (DYNAMIC-ICU): A prospective cohort study. Int J Nurs Stud. 2019;93:64-73

41. Leppink J, Paas F, Van der Vleuten CP, Van Gog T, Van Merrienboer JJ. Development of an instrument for measuring different types of cognitive load. Behav Res Methods. 2013;45(4):1058-72

42. Trogrlic Z, van der Jagt M, Lingsma H et al. Improved Guideline Adherence and Reduced Brain Dysfunction After a Multicenter Multifaceted Implementation of ICU Delirium Guidelines in 3,930 Patients. Crit Care Med. 2019;47(3):419-427

43. Dykes PC, Carroll DL, Hurley A et al. Fall prevention in acute care hospitals: a randomized trial. JAMA. 2010;304(17):1912-8

44. Xing J, Sun Y, Jie Y, Yuan Z, Liu W. Perceptions, attitudes, and current practices regards delirium in China: A survey of 917 critical care nurses and physicians in China. Medicine (Baltimore). 2017;96(39):e8028
