# Supplementary material for: Impact of Clinical Decision Support System Assisted prevention and management for Delirium on guideline adherence and cognitive load among Intensive Care Unit nurses (CDSSD-ICU): Protocol of a multicentre, cluster randomized trial
Source: PLoS One. 2023 Nov 28;18(11):e0293950. doi: 10.1371/journal.pone.0293950 (PMC10684021; doi:10.1371/journal.pone.0293950)
Supplement: Attachment — Submitted filename: Response to Reviewers.docx [file pone.0293950.s008.docx]

**Journal Requirements:**
Please review your reference list to ensure that it is complete and correct. If you have cited papers that have been retracted, please include the rationale for doing so in the manuscript text, or remove these references and replace them with relevant current references. Any changes to the reference list should be mentioned in the rebuttal letter that accompanies your revised manuscript. If you need to cite a retracted article, indicate the article’s retracted status in the References list and also include a citation and full reference for the retraction notice.
**Response:** *We reviewed the reference list to ensure that it is complete and correct.*

[Note: HTML markup is below. Please do not edit.]
**Reviewers' comments:**

**Comments to the Author**
1. Does the manuscript provide a valid rationale for the proposed study, with clearly identified and justified research questions?
The research question outlined is expected to address a valid academic problem or topic and contribute to the base of knowledge in the field.

Reviewer #2: Partly

2. Is the protocol technically sound and planned in a manner that will lead to a meaningful outcome and allow testing the stated hypotheses?
The manuscript should describe the methods in sufficient detail to prevent undisclosed flexibility in the experimental procedure or analysis pipeline, including sufficient outcome-neutral conditions (e.g. necessary controls, absence of floor or ceiling effects) to test the proposed hypotheses and a statistical power analysis where applicable. As there may be aspects of the methodology and analysis which can only be refined once the work is undertaken, authors should outline potential assumptions and explicitly describe what aspects of the proposed analyses, if any, are exploratory.

Reviewer #2: Yes

3. Is the methodology feasible and described in sufficient detail to allow the work to be replicable?
Descriptions of methods and materials in the protocol should be reported in sufficient detail for another researcher to reproduce all experiments and analyses. The protocol should describe the appropriate controls, sample size calculations, and replication needed to ensure that the data are robust and reproducible.

Reviewer #2: No

4. Have the authors described where all data underlying the findings will be made available when the study is complete?
The [PLOS Data policy](https://journals.plos.org/plosone/s/materials-and-software-sharing) requires authors to make all data underlying the findings described in their manuscript fully available without restriction, with rare exception, at the time of publication. The data should be provided as part of the manuscript or its supporting information, or deposited to a public repository. For example, in addition to summary statistics, the data points behind means, medians and variance measures should be available. If there are restrictions on publicly sharing data—e.g. participant privacy or use of data from a third party—those must be specified.

Reviewer #2: Yes

5. Is the manuscript presented in an intelligible fashion and written in standard English?
PLOS ONE does not copyedit accepted manuscripts, so the language in submitted articles must be clear, correct, and unambiguous. Any typographical or grammatical errors should be corrected at revision, so please note any specific errors here.

Reviewer #2: Yes

**Response:** *We provided more detailed information in the methodology to allow the work to be replicable, please see the detailed information in the Materials and methods, Supplementary file 3 (The Clinical Decision Support System Assisted Prevention and Management for Delirium).*

**6. Review Comments to the Author**
Reviewer #2: Dear authors,
I am very pleased that you have implemented my suggestions. There are still some ambiguities that are probably easy to implement.

Z.72 - 75: The cited literature by Han et al. 2021 describes communication via text messaging in an intensive care unit .
**Response:** *We replaced the cited literature by Han et al. 2021 as Ceballos-Vasquez et al:*

*Ceballos-Vasquez P, Rolo-Gonzalez G, Hernandez-Fernaud E, Diaz-Cabrera D, Paravic-Klijn T, Burgos-Moreno M. Psychosocial factors and mental work load: a reality perceived by nurses in intensive care units. Rev Lat Am Enfermagem. 2015;23(2):315-22. doi: 10.1590/0104-1169.0044.2557.*

Z.86 With regard to the development of the AI-AntiDelirium, you refer to another publication as well as to 5 steps of the development of the AI-AntiDelirium. Were the results of the development process published? If not, would a separate publication be interesting?

**Response:** *We appreciate the reviewer very much for your positive comments and interest in the development of the AI-AntiDelirium. Detailed information about the Development of an Artificial Intelligence-Assisted Prevention and Management for Delirium (AI-AntiDelirium) is under review in a peer-reviewed journal.*

Is the AI-AntiDelirium really artificial intelligence? Is it not rather a Clinical Decision Support System (CDSS) that acts on the basis of predefined IF-Then rules? You might want to reconsider the term Artificial Intelligence in the title and in the acronym "AI-AntiDelirium" and in other places.

**Response:** *Thanks a lot for your very helpful comment. As you suggested, we modified the Title as following:*

*Impact of* ***C****linical* ***D****ecision* ***S****upport* ***S****ystem Assisted Prevention and Management for* ***D****elirium on guideline adherence and cognitive load in* ***I****ntensive* ***C****are* ***U****nit nurses* ***(CDSSD-ICU)****: protocol of a multicenter, cluster randomized trial.*

*In addition, we revised the the acronym "AI-AntiDelirium" into "CDSS-AntiDelirium".*

203: The calculation of adherence is now more comprehensible. However, the adherence cannot be between 0 and 1 if the calculation of the formulas a, b, c is done according to a different systematic.

**Response:** *Thanks a lot for your kindly comment. We revised the calculation of adherence as following:*

*Complete adherence rate is recorded as 100%, and complete non-adherence rate is recorded as 0%. Adherence rate is between 0 and 100 according to the following formula.*

257: The description of the level of knowledge of the nurses rather belongs to the chapter Outcomes.

**Response:** *As you suggested, we moved the level of knowledge of the nurses into the Outcome measures section.*

243: You assume that the recruitment of nurses will be completed by the end of June 2023. What is the current status of recruitment? Are changes to the protocol still feasible?
**Response:** *The current status of recruitment is finished, between November 2022 and June 2023, 38 nurses (mean [SD] age, 33.29 [0.88] years; 33 [86.8%] female) are included in the intervention group, and 42 nurses (mean [SD] age, 33.52 [0.79] years; 39 [92.9%] female) are recruited in the control group. Changes to the protocol are still feasible, we continue to collect the outcomes among recruiting ICU nurses.*
