# Supplementary material for: Impact of Clinical Decision Support System Assisted prevention and management for Delirium on guideline adherence and cognitive load among Intensive Care Unit nurses (CDSSD-ICU): Protocol of a multicentre, cluster randomized trial
Source: PLoS One. 2023 Nov 28;18(11):e0293950. doi: 10.1371/journal.pone.0293950 (PMC10684021; doi:10.1371/journal.pone.0293950)
Supplement: Attachment — Submitted filename: Response to Reviewers.docx [file pone.0293950.s009.docx]

**Additional Editor Comments:**

The paper has again been reviewed and the reviewer is mostly happy with the manuscript.

However, before publication,there are three more aspects to be addressed:

(1) Most importantly, please apply another language check preferably by a professional language editor as there are still a number of inadequate expressions and language use is often incorrect.

**Response:** *Thanks a lot for your comment. As you suggested, we polished the manuscript by a professional language expert.*

(2) In l.139, you state that “Investigators who will enroll participants are not made aware of the randomization list.” Please be clearer about this process. As “post-randomization recruitment bias” is a common risk of bias in cluster randomized studies, it is important to know, who is recruiting patients and determining their eligibility.

**Response:** *We provided more information about this process, as following:*

*Investigators who will enroll participants will not be made aware of the randomization list. Once the ICUs (cluster) that will take part in the study have been determined, randomization will be performed, considering the size of each ICU and the type of disease treated. The following criterion will be considered in this process: whether the center is classified as having a high incidence of delirium. Investigators will recruit participants following predefined inclusion and exclusion criteria.*

(3) Under “Patient and public involvement” (l.347ff.), please be more specific. Information is not involvement. So if there is no active role in the sense of PPI, please state this clearly.

**Response:** *We provided more detailed information in the “Patient and public involvement”, as following:*

*Patients and/or the public (including clinical professionals) will be involved in the study design, or conduct, or reporting of this research. A participatory workshops carried out in October 2022 focused on collecting their opinions and solving problems in the design of the protocol.*
